# The GATA-Type Transcription Factor Csm1 Regulates Conidiation and Secondary Metabolism in *Fusarium fujikuroi*

**DOI:** 10.3389/fmicb.2017.01175

**Published:** 2017-06-26

**Authors:** Eva-Maria Niehaus, Julia Schumacher, Immo Burkhardt, Patrick Rabe, Martin Münsterkötter, Ulrich Güldener, Christian M. K. Sieber, Jeroen S. Dickschat, Bettina Tudzynski

**Affiliations:** ^1^Institut für Biologie und Biotechnologie der Pflanzen, Westfälische Wilhelms-Universität Münster Münster, Germany; ^2^Kekulé-Institut für Organische Chemie und Biochemie, Rheinische Friedrich-Wilhelms-Universität Bonn Bonn, Germany; ^3^Institute of Bioinformatics and Systems Biology, German Research Center for Environmental Health (GmbH), Helmholtz Zentrum München Neuherberg, Germany; ^4^Department of Genome-Oriented Bioinformatics, Wissenschaftszentrum Weihenstephan, Technische Universität München Freising, Germany; ^5^Department of Energy, Joint Genome Institute, Walnut Creek CA, United States

**Keywords:** *Fusarium fujikuroi*, GATA transcription factor, conidiation, secondary metabolism, gene expression, NsdD, Csm1, STC

## Abstract

GATA-type transcription factors (TFs) such as the nitrogen regulators AreA and AreB, or the light-responsive TFs WC-1 and WC-2, play global roles in fungal growth and development. The conserved GATA TF NsdD is known as an activator of sexual development and key repressor of conidiation in *Aspergillus nidulans*, and as light-regulated repressor of macroconidia formation in *Botrytis cinerea.* In the present study, we functionally characterized the NsdD ortholog in *Fusarium fujikuroi*, named Csm1. Deletion of this gene resulted in elevated microconidia formation in the wild-type (WT) and restoration of conidiation in the non-sporulating velvet mutant Δ*vel1* demonstrating that Csm1 also plays a role as repressor of conidiation in *F. fujikuroi*. Furthermore, biosynthesis of the PKS-derived red pigments, bikaverin and fusarubins, is de-regulated under otherwise repressing conditions. Cross-species complementation of the Δ*csm1* mutant with the *B. cinerea* ortholog *LTF1* led to full restoration of WT-like growth, conidiation and pigment formation. In contrast, the *F. fujikuroi CSM1* rescued only the defects in growth, the tolerance to H_2_O_2_ and virulence, but did not restore the light-dependent differentiation when expressed in the *B. cinerea* Δ*ltf1* mutant. Microarray analysis comparing the expression profiles of the *F. fujikuroi* WT and the Δ*csm1* mutant under different nitrogen conditions revealed a strong impact of this GATA TF on 19 of the 47 gene clusters in the genome of *F. fujikuroi*. One of the up-regulated silent gene clusters is the one containing the sesquiterpene cyclase-encoding key gene *STC1.* Heterologous expression of *STC1* in *Escherichia coli* enabled us to identify the product as the volatile bioactive compound (–)-germacrene D.

## Introduction

Filamentous fungi produce a diverse array of low-molecular-mass compounds known as secondary metabolites (SMs). They are of enormous interest to humankind due to their pharmaceutical activities (e.g., as antibiotics or immunosuppressants) as well as their toxic properties (mycotoxins). The sequencing of an increasing number of fungal genomes has greatly facilitated the *in silico* identification of potential SM biosynthetic genes and gene clusters. Especially, clusters containing modular polyketide synthase (PKS) or non-ribosomal peptide synthetase (NRPS) genes are easy to detect by scanning the genome for genes that encode enzymes with conserved characteristic domains, e.g., the “adenylation (A)” and “condensation (C)” domains of NRPSs (Weber and Kim, 2016). Unfortunately, many of these SM gene clusters either remain “silent" or weakly expressed under standard experimental conditions and consequently many of their products are still unknown (Brakhage, 2013). Therefore, a better understanding of the complex regulatory network that modulates the expression of SM biosynthetic genes is of great importance to overcome the silencing of cryptic gene clusters and to discover new bioactive compounds.

Several clusters contain cluster-specific transcription factors (TFs), mostly positive acting Zn(II)_2_Cys_6_ zinc binuclear type TFs, that regulate expression of the adjacent cluster genes responsible for biosynthesis of the respective metabolite. Over-expression of these pathway-specific TFs may elevate cluster gene expression and, in the case of silent clusters, can result in their activation (Brakhage, 2013; Niehaus et al., 2014). Global regulators that are able to integrate cellular responses to environmental cues, such as nutrient availability, pH and light are also able to regulate SM clusters (Yin and Keller, 2011). Examples of such global regulators are: PacC, the key player of fungal pH regulation (Keller et al., 1997; Wiemann et al., 2009); AreA and AreB, the two GATA-type TFs involved in nitrogen regulation (Mihlan et al., 2003; Michielse et al., 2014; Pfannmüller et al., 2017) and WC-1 and WC-2 (white collar), the GATA factors involved in regulation of blue light responses (Dunlap, 1999; Estrada and Avalos, 2008; Canessa et al., 2013).

It has long been noted that biosynthesis of SMs is often associated with cell differentiation (Calvo et al., 2002). The conserved Velvet complex is one of the best-known examples of a developmental regulator. It consists of at least three components, i.e., VeA, VelB, and LaeA (Bok and Keller, 2004; Bayram et al., 2008) and links secondary metabolism and sexual/asexual differentiation processes in many fungi. In *Aspergillus nidulans*, VeA is a light-dependent activator of penicillin and sterigmatocystin biosynthesis and a negative regulator of conidiation (Kato et al., 2003). In *Fusarium fujikuroi*, the VeA homolog Vel1 activates gibberellin biosynthesis, but represses bikaverin biosynthesis in a light-independent manner. In contrast to *A. nidulans*, Vel1 promotes the formation of conidia and represses sexual development (Wiemann et al., 2010). Another global regulator that regulates sexual and/or asexual reproduction and production of SMs is the conserved GATA TF NsdD in *A. nidulans* and *Aspergillus fumigatus*. Orthologous proteins are Sub-1 in *Neurospora crassa*, Pro44 in *Sordaria macrospora*, and Ltf1 in *Botrytis cinerea* (Han et al., 2001; Grosse and Krappmann, 2008; Nowrousian et al., 2012; Schumacher et al., 2014). This GATA TF acts as developmental regulator in all these species. A link between conidiation on the one hand, and secondary metabolism on the other, has been observed in *A. nidulans* and *B. cinerea*, whereas additional functions involving reactive oxygen species (ROS) homoeostasis, light responses, and virulence have been shown in the plant pathogen *B. cinerea* (Schumacher et al., 2014).

In the current study, we analyzed the impact of the NsdD ortholog in *F. fujikuroi* that is a phytopathogenic fungus causing bakanae disease of rice seedlings due to its ability to produce large amounts of gibberellins, a class of phytohormones (Bömke and Tudzynski, 2009). However, heavily infected seedlings can also be stunted and can show severe crown and root rot. The type of symptoms and severity of disease depends on the fungal isolate and are thought to be affected by the proportions of gibberellins and other SMs such as fusaric acid (Smith and Dilday, 2003). However, this assumption has never been proved experimentally.

Recent genome sequencing revealed 47 potential SM biosynthetic gene clusters (Wiemann et al., 2013; Niehaus et al., 2016a). The majority of these gene clusters are silent under standard laboratory conditions, and as in other fungi, no metabolic function could be assigned. Current research has shown that 15 of these gene clusters could be linked to their respective products, but only seven of them contain TF-encoding genes (Wiemann et al., 2009; Studt et al., 2012; Brock et al., 2013; Niehaus et al., 2014; von Bargen et al., 2015; Burkhardt et al., 2016; Janevska et al., 2016; Niehaus et al., 2016b; Rösler et al., 2016; Studt et al., 2016). One strategy to activate those gene clusters would be the genetic manipulation of global regulators, such as NsdD. This GATA-type TF has been shown not only to affect sexual and asexual reproduction but also secondary metabolism in *Aspergillus* spp. (Lee et al., 2016).

Here, we provide a comprehensive functional analysis of the NsdD ortholog in *F. fujikuroi.* We show that the TF acts as repressor of conidiation and as global regulator of secondary metabolism and named it therefore Csm1. Cross-species complementation of the *CSM1* deletion mutant with *LTF1* from *B. cinerea* fully restored asexual development and SM production, whereas *CSM1* from *F. fujikuroi* only partially restored wild-type (WT) features in *B. cinerea* Δ*ltf1* mutants. A genome-wide transcriptome analysis under different nitrogen conditions revealed a large number of Csm1-dependent genes. Among them are many SM biosynthetic genes. One of the up-regulated key enzyme-encoding genes is *STC1* encoding a sesquiterpene cyclase with unknown function. Heterologous expression of *STC1* in *Escherichia coli* resulted in the identification of its product.

## Materials and Methods

### Fungal and Bacterial Strains

Strain IMI 58289 (International Mycological Institute, Kew, United Kingdom) is a gibberellin-producing WT strain of *F. fujikuroi*. The *F. fujikuroi* Δ*vel1* mutant (Wiemann et al., 2010) was used to generate a double Δ*vel1/*Δ*csm1* mutant. *B. cinerea* strain B05.10 is an isolate from *Vitis* (Büttner et al., 1994); its derivative Δ*ltf1* was used as the recipient for cross-species complementation approach (Schumacher et al., 2014). *E. coli* strain Top10F’ (Invitrogen, Groningen, The Netherlands) was used for plasmid propagation. The uracil-auxotrophic *Saccharomyces cerevisiae* strain FGSC 9721 (FY 834) was provided by the Fungal Genetics Stock Center (Kansas State University) and used for yeast recombination cloning.

### Culture Conditions

*Fusarium fujikuroi*: For liquid cultures, strains were pre-cultured for 3 days in 300-mL Erlenmeyer flasks with 100 mL Darken medium (Darken et al., 1959) on a rotary shaker at 180 rpm at 28°C. 500 μL of the cultures were then used to inoculate 100 mL of ICI (Imperial Chemical Industries, United Kingdom) medium (Geissman et al., 1966) containing either 6 mM glutamine, 60 mM glutamine, 6 mM, 12 mM or 120 mM NaNO_3_. For SM analysis, the strains were grown for 7 days on a rotary shaker at 28°C in darkness. The supernatants of three biological replicates were filtered through a 0.45 μm sterile filter and analyzed via HPLC-DAD. For gene expression analyses, the mycelia were harvested after 2 and 4 days to study the expression of bikaverin and fusarubin biosynthetic genes, washed with deionized water and lyophilized. For plate assays, strains were cultivated on solid complete medium (CM) (Pontecorvo et al., 1953), synthetic Czapek-Dox (CD) agar (Sigma–Aldrich, Germany), and V8 agar (160 mL V8 vegetable juice, 3 g/L CaCO_3_, 20 g/L agar). For stress resistance assays, 40 mM hydrogen peroxide (H_2_O_2_, Sigma–Aldrich, Germany) was added. Cultures were incubated at 20°C under white light (12 h light/12 h darkness) or in constant darkness. For quantification of conidiation, the strains (three biological replicates) were grown for 14 days at room temperature on V8 agar under light/dark conditions.

*Botrytis cinerea*: Strains were cultured on CM agar at 20°C under light/dark conditions for induction of conidiation and in constant darkness for induction of sclerotial development. Virulence was tested on primary leaves of living *Phaseolus vulgaris* plants by using plugs of vegetative mycelia.

*Saccharomyces cerevisiae*: The strains were propagated in YPD (yeast extract-peptone-dextrose) medium. For selection of URA+ strains, SD-uracil medium was used.

*Streptomyces flavochromogenes* was cultured in Gym 65 liquid medium (glucose 4.0 g, yeast extract 4.0 g, malt extract 4.0 g, 1 L water, pH 7.2) for 5 days at 28°C for isolation of genomic DNA.

For fluorescence microscopy, strains were first cultivated for 3 days on a CM agar plate. After that the mycelium was shaken in an Eppendorf tube for 24 h in ICI medium supplemented with 6 mM NaNO_3_ or 6 mM glutamine. The supernatant was used for microscopy.

### Standard Molecular Methods

Fungal genomic DNA, plasmid DNA, and total RNA were prepared as described previously (Cenis, 1992; Wiemann et al., 2012). Isolation of plasmid DNA from *S. cerevisiae* was done using the kit Zymoprep Yeast Plasmid Miniprep II (Zymo Research, Irvine, CA, United States). For northern blot analyses (two biological replicates), 20 μg of total RNA were separated in 1% (w/v) denaturating agarose gels. Upon transfer of the separated RNA to nylon membranes (Nytran^TM^ SPC, Whatman, Sanford, FL, United States), the blots were hybridized with ^32^P-labeled probes using the random oligomer-primer method and membranes were hybridized as described previously (Sambrook et al., 1989). The following probes were amplified with the following primers: *BIK2* (bik2-F/bik2-R), *FSR2* (fsr2-F/fsr2-R), *CSM1* (csm1-F/csm1-R), *LTF1* (*LTF1*-F/*LTF1*-R), *LTF2* (*LTF2*-F/*LTF2*-R), *PKS13* (*PKS13*-F/*PKS13*-R), and CCG1 (*CCG1*-F/*CCG1*-R) (Supplementary Table S1).

PCR reactions were done by use of the high-fidelity DNA polymerase Phusion (Finnzymes, Finland) for cloning purposes and the BioTherm Taq DNA Polymerase (GeneCraft, Germany) for diagnostic applications. Replacement fragments and expression constructs were assembled in *S. cerevisiae* by exploiting its homologous recombination machinery (Colot et al., 2006; Schumacher, 2012). Sequencing of DNA fragments was performed with the Big Dye Terminator v3.1 sequencing kit (Applied Biosystems, United States) in an ABI Prism capillary sequencer (model 3730; Applied Biosystems). For sequence analysis, the program package DNA-Star (Madison, WI, United States) was used.

Generation of protoplasts and transformation of *F. fujikuroi* were carried out according to (Tudzynski et al., 1999). Regeneration of transformed protoplasts was performed for 4–5 days at 28°C in a regeneration medium (0.7 M sucrose, 0.05% yeast extract) containing either 100 mg/mL nourseothricin (Werner-Bioagents, Jena, Germany) or 100 mg/mL hygromycin (Calbiochem, Darmstadt, Germany). Generation and transformation of protoplasts and the selection of nourseothricin-resistant transformants in *B. cinerea* was accomplished as described previously (Schumacher, 2012).

### Vector Cloning and Generation of Mutants

For generation of *CSM1* deletion mutants, the 1-kb-long flanking regions were amplified using primer pairs csm1-3F/3R and csm1-5F/5R (Supplementary Table S1 and Figure S1). The flanks and the hygromycin (derived from pCSN44) or the nourseothricin (derived from pZPnat1) resistance cassette were cloned into the linearized shuttle vector pRS426 by yeast recombinational cloning (Colot et al., 2006; Schumacher, 2012) generating the vectors pΔ*csm1*_hph and pΔ*csm1*_nat1, respectively. The Δ*csm1*_hph fragment was transformed into the WT yielding Δ*csm1* mutants (for Southern blot see Supplementary Figure S1B). The Δ*csm1*_nat1 fragment was transformed into the Δ*vel1* mutant generating double Δ*vel1*/Δ*csm1* mutants.

Constructs with different *CSM1* versions for the targeted integration at the *CSM1* locus (*in loco* integration) were generated as follows (Supplementary Figures S1A,D): PCR fragments of the coding regions of *CSM1*, *LTF1* or *GFP* were co-transformed with T*_GLUC_* (terminator of a glucanase-encoding gene of *B. cinerea*) and the *Sph*I/*Sma*I-restricted pΔcsm1_nat1 comprising the *CSM1*-flanking regions and the nourseothricin resistance cassette. T*_GLUC_* (0.500 kb) was amplified by using primers Glu-term-F2 and Tgluc-nat1-R and genomic DNA of *B. cinerea* as template; the codon-optimized *GFP* (0.780 kb) using primers *oGFP*-F and *oGFP*-NotI-R1 and pNDN-OGG (Schumacher, 2012) as template. (1) Constructs for the expression of *CSM1(-GFP)* (complementation): coding region of *CSM1* (1.395 kb) was amplified from genomic DNA of *F. fujikuroi* with primer pairs *CSM1*-com-F/*CSM1*-T*GLUC*-R and *CSM1*-com-F/*CSM1*-com-*GFP*-R for generating p*CSM1*^C^ *^CSM*1*^* and p*CSM1::GFP*, respectively. (2) The construct for the expression of *LTF1* (cross-species complementation): coding region of *LTF1* (1.640 kb) was amplified from genomic DNA of *B. cinerea* with primer pair CSM1-comp-5′-*LTF1*-F/*LTF1*-Tgluc-R for generating p*CSM1*^C^ *^LTF*1*^*. (3) Constructs for the expression of a mutated *CSM1(-GFP)* variants [three nucleotides of the motif 5′-CGCCAGTCGCTGCCCTCAATC-3′ (RQSLPSI) were changed resulting in the following sequence: 5′-GGCCAGGCGCTGCCCGCAATC-3′ (GQALPAI)]. The coding region of *CSM1* was amplified in two parts from genomic DNA of *F. fujikuroi* by using primer pairs *CSM1*-5F/*CSM1*-mut-R (0.192 kb) and *CSM1*-mut-F/*CSM1*-T*GLUC*-R (for p*CSM1*^MUT^) and *CSM1*-mut-F/*CSM1*-com-*GFP*-R (for p*CSM1*^MUT^::*GFP*) (1.232 kb). Prior to transformation of the Δ*csm1* mutant, the plasmids were linearized with *Apa*I. Targeted integration at the *CSM1* locus resulting in the replacement of the hygromycin resistance cassette, was detected by diagnostic PCR using the primer combination nat-hiR/*CSM1*-3R-diag in independent transformants (*CSM1*^C^ *^CSM*1*^*, Δ*csm1*/*CSM1*::*GFP*, *CSM1*^C^ *^LTF*1*^*, Δ*csm1*/*CSM1*^MUT^::*GFP*) (Supplementary Figures S1C,E).

For expression of *CSM1* in *B. cinerea* under control of the *LTF1* promoter, the gene was introduced in the *LTF1* locus by replacing the hygromycin resistance cassette in the Δ*ltf1* mutant (Supplementary Figure S2A). *CSM1* (1.398 kb) was amplified with primers *CSM1-ltf1*-F and *CSM1-ltf1*-R using genomic DNA of *F. fujikuroi* as template, and assembled with the *Hin*dIII/*Eco*RI-digested plasmid pLTF1-GFP^iL^ (Schumacher et al., 2014) yielding pltf1^C^ *^CSM*1*^*. The plasmid was linearized with *Apa*I prior to the transformation. Targeted integration at the *LTF1* locus was detected using primer combinations *LTF1*-A-hi5F/*CSM1*-sR1 and *LTF1*-hi3R/*nat1*-hiR. Single spore isolates of the transformants were screened for the absence of the Δ*ltf1* allele by using the primer combination *LTF1*-A-hi5F/*hph*-hiF. In summary, two independent homokaryotic *LTF1*^C^ *^CSM*1*^* mutants with identical phenotypes were obtained (Supplementary Figures S2B, S5).

For the construction of plasmids for heterologous expression in *E. coli*, the genes encoding Csm1 in *F. fujikuroi* and the geosmin synthase in *S. flavochromogenes* (accession number: WP_030314776) were amplified from the respective genomic DNA with the primer pair Invitro_STC1_F/Invitro_STC1_R and PR086f_WP030314776/PR086r_WP030314776, respectively. For homologous recombination in yeast, the amplified gene was elongated in a second PCR with elongated primers PR088f_WP030314776 and PR088r_WP030314776 (Supplementary Table S1). This PCR product and the *Xho*I/*Pvu*II-linearized vector pYE-Express were used for homologous recombination in *S. cerevisiae* using the LiOAc/SS carrier DNA protocol (Dickschat et al., 2014). Electroporation of the isolated plasmids in *E. coli* BL 21 cells, cultivation on 2YT agar plates at 37°C overnight and selection of a single colony resulted in the plasmid pYE_WP_030314776 whose sequence was confirmed by sequencing.

### Microarray Analysis

The *F. fujikuroi* microarray was designed by Roche NimbleGen Systems (Madison, WI, United States) as described previously (Wiemann et al., 2013). Microarray hybridizations were performed at Arrows Biomedical (Münster, Germany) and RNA quality was checked using Agilent Bioanalyzer 2100 and RNA Nano 6000 Lab-Chip Kit (Agilent Technologies).

Expression data were analyzed as described before (Wiemann et al., 2013). Genes with an absolute log_2_-fold change above one or below minus one and an adjusted *P*-value (FDR) below 0.05, based on biological duplicates, were regarded as significantly differentially expressed. The expression datasets are available in the Gene Express Omnibus (GEO) repository^1^.

To explore functional distributions of specific regulated gene sets the Functional Catalog (FunCat) (Ruepp et al., 2004) was used to identify biological processes. We applied Fisher’s exact test (Fisher, 1922) to determine statistically overrepresented functional categories in differentially expressed gene sets. The retained *P*-values were adjusted using Bonferroni procedure. Tested categories with an adjusted *P*-value below 0.05 were regarded as significantly overrepresented in the gene set.

### Heterologous Expression of *STC1* in *E. coli* and Enzyme Incubations

*Escherichia coli* BL 21 transformants were inoculated in a 2YT liquid pre-culture containing kanamycin (50 mg/L) over night. For protein isolation, the pre-culture was used to inoculate 500 mL 2YT liquid cultures containing kanamycin (50 mg/L). After cultivation of *E. coli* BL 21 to an OD_600_ = 0.6 at 37°C and 160 rpm, the cells were cooled to 20°C for 30 min followed by addition of IPTG (0.4 mM). The culture was incubated at 18°C and 160 rpm overnight. Cells were harvested by centrifugation at 4°C and 8000 rpm for 30 min. The pellet was resuspended in 10 mL binding buffer (20 mM Na_2_HPO_4_, 0.5 M NaCl, 20 mM imidazole, 1 mM MgCl_2_, pH 7.0). The cells were disrupted by ultra-sonication on ice for 6 × 30 s. The cell debris was removed by repeated centrifugation (2 min × 10 min) at 4°C and 11,000 rpm to yield the soluble enzyme fractions. Protein purification was performed by Ni^2+^-NTA affinity chromatography with Ni^2+^-NTA superflow (Qiagen) using binding buffer and elution buffer (2 × 10 mL; 20 mM Na_2_HPO_4_, 0.5 M NaCl, 500 mM imidazole, 1 mM MgCl_2_, pH 7.0). The soluble enzyme fractions were checked by SDS–PAGE. The pure fractions were used for incubation experiments with the natural substrate FPP (0.5 mg and 0.25 mg/mL). The incubation experiment of the *S*. *flavochromogenes* geosmin synthase was done at 28°C for 3 h and stopped by extraction with cyclohexane (0.5 mL). The STC1 incubation experiment was conducted at 28°C overnight and the mixture subsequently extracted with *n*-hexane (0.5 mL). The extracts were dried over MgSO_4_ and directly analyzed by GC/MS.

### GC/MS Analysis

Standard GC/MS analyses were carried out with a HP 7890B gas chromatograph connected to a HP 5977A inert mass detector fitted with parameters were (1) inlet pressure, 77.1 kPa, He 23.3 mL/min, (2) injection volume, 2 μL, (3) transfer line, 250°C, and (4) electron energy 70 eV. A HP5-MS column (Agilent, 30 m length, 0.25 mm diameter, 0.50-μm film) was used and the GC was programmed as follows: 5 min at 50°C increasing at 5°C min^-1^ to 320°C, and operated in split mode (10:1, 60 s valve time). The carrier gas was He at 1 mL/min. For determination of the absolute configuration of germacrene D, the extracts of incubation experiments and mixtures thereof were subjected to GC/MS analysis on the same gas chromatograph and mass detector using a Cyclosil-B column (Agilent, 30 m length, 0.25 mm diameter, 0.25 μm film). A suitable GC Program for separating (+)- and (-)-germacrene D (5 min at 70°C increasing at 2°C min^-1^ to 170°C and increasing at 10°C/min to 200°C, operated in splitless mode) was determined by separating the enantiomeric germacrene D mixture from commercially available essential oil of *Solidago canadensis* (Pranarom, Belgium).

### Chemical Analysis of Secondary Metabolites

For analyses of the SMs, strains were grown in submerged cultures as described above. After 7 days, mycelia were removed from the culture by filtration through Mirachloth (Calbiochem, Merck KGaA, Darmstadt, Germany). Small particulates were removed from the culture filtrates using 0.45 μm syringe filters (BGB^®^, Schloßböckelheim, Germany) which were then analyzed by high-pressure liquid chromatography with a diode array detector [HPLC-DAD; Hitachi Chromaster LC equipped with a 250 mm × 4.60 mm i.d., 5 μm, Gemini^®^ C_18_ with a 4 mm × 3 mm Gemini^®^ C_18_ guard column (Phenomenex, Aschaffenburg, Germany)], a with 5160 pump, 5260 autosampler, 5310 column oven, and 5430 DAD (VWR International GmbH, Darmstadt, Germany). For data analyses, the software EZChrom Elite (VWR, Darmstadt, Germany) was used.

### Fluorescence Microscopy

Microscopy was performed using the Axio Imager.M2 (Carl Zeiss MicroImaging GmbH, Jena, Germany). Fluorescence of GFP was detected using filter set 38 (excitation BP 470/40, beam splitter FT 495, emission BP 525/50). Images were captured using equal exposure times and applying the AxioCam MRm (Carl Zeiss MicroImaging GmbH, Jena, Germany). Nuclei were stained with Hoechst 33342 (Sigma–Aldrich, Chemie GmbH, Steinheim, Germany) in a 1:1000 dilution in McIlvaine Buffer pH 7.2 (Kangatharalingam and Ferguson, 1984), incubated for 1 min and visualized with filter set 49 DAPI shift free (excitation G 365, beam splitter FT 395, emission BP 445/50). All images were processed with the AxioVision Rel. 4.8 software (Carl Zeiss MicroImaging GmbH, Jena, Germany).

## Results

### Identification of the NsdD Ortholog in *F. fujikuroi*

**FIGURE 1:**
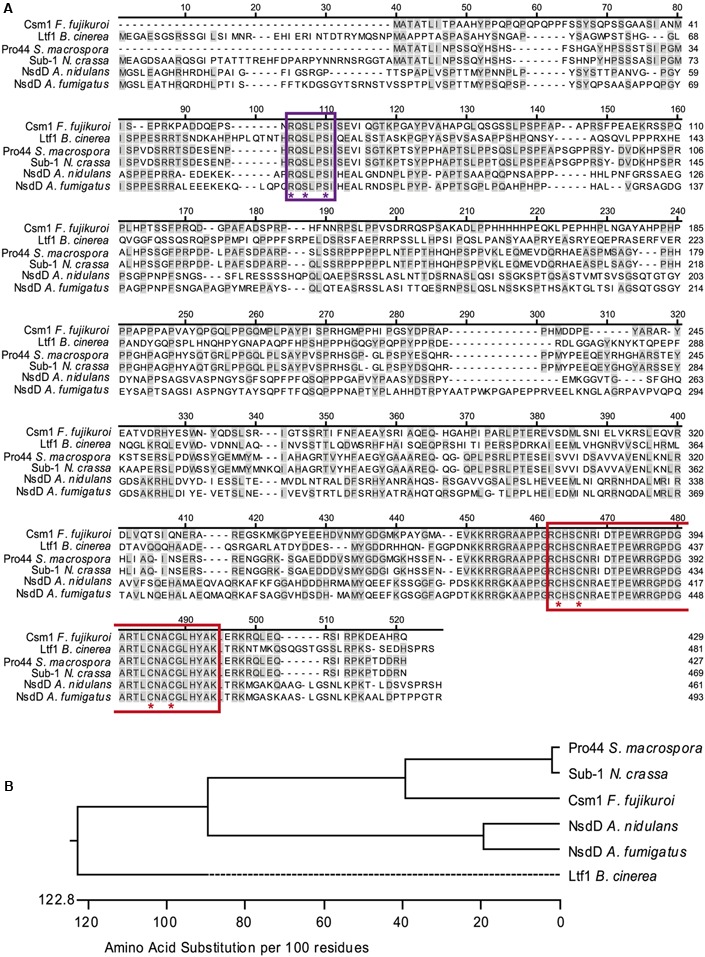
Sequence alignment and phylogenetic analysis of *CSM1*. **(A)** Sequence alignment of Csm1 and homologous protein sequences from different filamentous fungi, generated using ClustalW alignment. Sequences used are *Fusarium fujikuroi* Csm1 (429 aa, FFUJ_07383), *Botrytis cinerea* Ltf1 (481 aa, B0510_3555), *Neurospora crassa* Sub-1 (466 aa, NCU01154.7), *Sordaria macrospora* Pro44 (427 aa, SMAC_03223), *Aspergillus nidulans* NsdD (461 aa, An3152), and *A*. *fumigatus* NsdD (493 aa, Afu3g13870). Amino acid residues shared with Csm1 are colored in gray. The GATA zinc finger domain (PF00320) is indicated in red and the conserved cysteine residues are marked by red asterisks. The uncharacterized conserved motif RQSLPSI is marked by violet box. Violet asterisks indicate the three amino acid changes in Csm1^MUT^. **(B)** Phylogenetic tree of Csm1 orthologs based on amino acid substitutions according to the alignment shown in **(A)**.

BlastP analyses against the complete *F. fujikuroi* genome database using the protein sequence of *A. nidulans* NsdD (AAB16914) as query predicted the product of gene *FFUJ_07383* as ortholog named *CSM1*. The coding region is 1,393 bp long and interrupted by two introns that were confirmed by sequencing the cDNA. The gene is located on chromosome 5 and flanked by unusual large non-coding regions (19 kb up- and 6.8 kb downstream). The alignment of protein sequences of Csm1, NsdD, Pro44, Ltf1, and Sub-1 showed low levels of overall sequence similarity (∼50% with Pro44 and Sub-1 and ∼30% with Ltf1 and NsdD) of Csm1 with the other fungal orthologs (**Figure 1A**). A high level of sequence similarity was found only for the C-terminal GATA zinc finger domain and a second highly conserved sequence motif RQSLPSI near the N-terminus which is present in all NsdD orthologs, but not in other fungal GATA-type TFs such as AreA, AreB, WC-1, and WC-2 (data not shown). Its potential importance for the protein function has not yet been studied. Phylogenetic analysis confirmed the closer relationship of Csm1 to the orthologs of two other members of the perithecium-forming class *Sordariomycetes*, *N. crassa*, and *S. macrospora*, compared to Ltf1 from *B. cinerea*, a member of the apothecium-forming class *Leotiomycetes*, and NsdD from *A. nidulans*, a member of the cleistothecium-forming class *Eurotiomycetes* (**Figure 1B**).

### Csm1 Has an Impact on Growth, Conidiation, Stress Tolerance and Pigmentation

**FIGURE 2:**
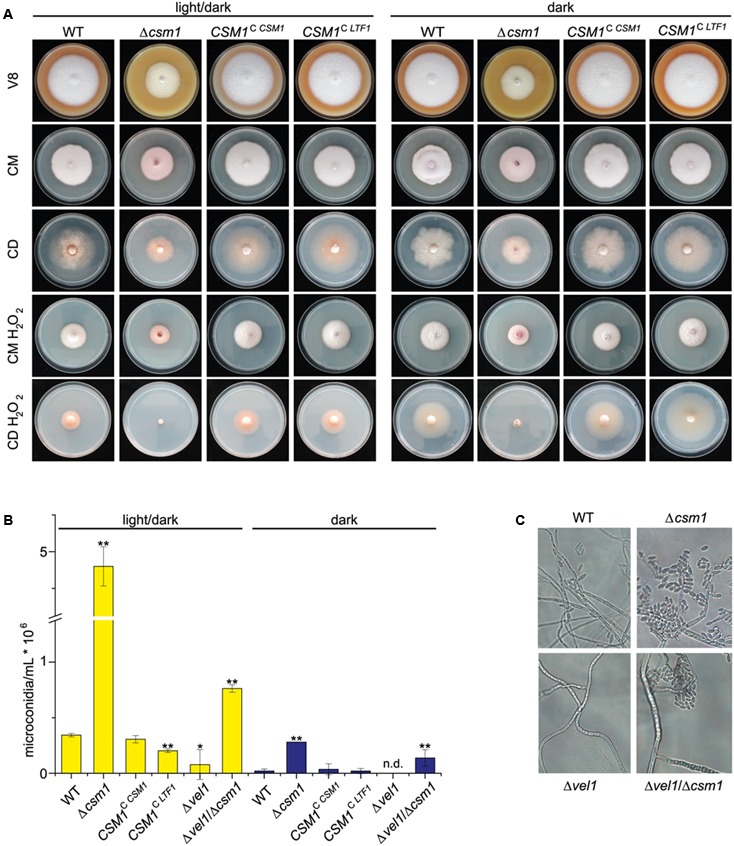
Deletion of *CSM1* affects colony growth and conidiation. **(A)** Colony morphology on different solidified media: V8 (vegetable juice medium), CM (complete medium), CD (minimal medium), and CM and CD supplemented with 40 mM H_2_O_2_. **(B)** Number of microconidia produced in light/dark (12h/12h) and in the dark. Mean values and standard deviations shown derived from three biological replicates. Statistical tests (*t*-test) revealed significant differences (^∗^*p* < 0.05 and ^∗∗^*p* < 0.001). **(C)** Microscopic observation of microconidia formation by wild-type (WT), Δ*csm1*, Δ*vel1* and Δ*vel1*/Δ*csm1* strains on V8 agar.

To study the function of Csm1 the gene was deleted in *F. fujikuroi*. In addition, complementation strains (*CSM1*^C^ *^CSM*1*^*) were generated by re-introduction of the WT copy into the native gene locus of the deletion mutant. The strains were grown on solid V8, CM, and synthetic CD agar for 6 days in darkness and in light (12 h light/12 h dark). The deletion mutants showed slightly reduced radial growth rates and reduced aerial hyphae formation compared to WT, whereas the complemented strain showed WT-like growth characteristics (**Figure 2A**).

To study the potential role of Csm1 to facilitate resistance to ROS, as shown for Ltf1 in *B. cinerea*, the strains were grown on solidified CM and minimal agar (CD agar) media containing 40 mM hydrogen peroxide (H_2_O_2_) at 20°C at both light conditions. Under these stress conditions, growth rates of the WT and the Δ*csm1* mutant were similarly affected on CM agar. However, the growth of the mutant was more reduced on minimal medium in the dark and almost totally abolished under light/dark conditions (**Figure 2A**). The complemented strain exhibited the WT phenotype.

One of the most obvious features of the Δ*csm1* mutant was the ability of the mutant to produce about 10- to 15-fold more microconidia than the WT on V8 agar under inducing light/dark conditions, but also in the dark (**Figure 2B**). The complemented strain produced WT-like numbers of microconidia. Previously we have shown that the Δ*vel1* mutant can no longer produce significant numbers of microconidia (Wiemann et al., 2010). To show whether the effect of the *VEL1* deletion can be overcome by the hypersporulation phenotype caused by the Δ*csm1* mutation, we generated a double Δ*vel1*/Δ*csm1* mutant. This mutant produced twofold more conidia than the WT and about 20% microconidia compared to the Δ*csm1* mutant indicating that the deficiency of the Δ*vel1* mutant to sporulate can be overcome by deleting *CSM1* (**Figures 2B,C**).

**FIGURE 3:**
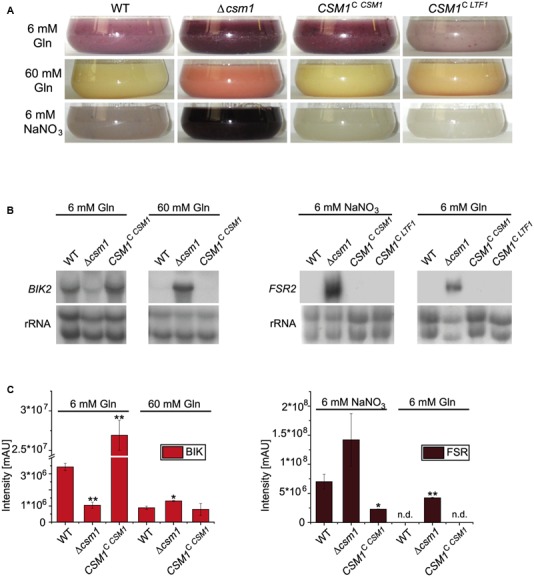
Biosynthesis of the polyketide synthase (PKS)-derived red pigments bikaverin and fusarubins. **(A)** Production of red pigments under different nitrogen and pH conditions. WT and mutant strains were grown for 2 days in synthetic Imperial Chemical Industries (ICI) medium with low (6 mM) and high (60 mM) amounts of glutamine (Gln, resulting in an acidic pH) and under low nitrogen alkaline conditions (6 mM NaNO_3_). **(B)** Northern blot analysis for the bikaverin (*BIK2*) and fusarubin (*FSR2*) biosynthetic genes. **(C)** Product yields of bikaverin and fusarubins. For northern blot and product analyses, strains were grown for 48 h and 7 days, respectively, in ICI medium with either 6 or 60 mM Gln (bikaverin, BIK) and 6 mM NaNO_3_ and 6 mM Gln (fusarubins, FSR). Statistical tests (*t*-test) revealed significant differences (^∗^*p* < 0.05 and ^∗∗^*p* < 0.001); n.d., not detected.

Another marked phenotype of the Δ*csm1* mutant is the altered pigmentation in axenic culture (**Figure 3A**). *F. fujikuroi* produces two PKS-derived red pigments, bikaverin and fusarubins. Bikaverin biosynthetic genes are only expressed under low nitrogen acidic conditions (6 mM glutamine) in a PacC-dependent manner (Wiemann et al., 2009). Furthermore, the GATA TF AreB was recently shown to act as a strong repressor of bikaverin gene expression (Pfannmüller et al., 2017). In contrast, the perithecial pigments fusarubins are produced under low nitrogen and alkaline conditions (6 mM NaNO_3_) (Studt et al., 2012). The Δ*csm1* mutant shows a more intense coloration in media with 6 mM glutamine and 6 mM NaNO_3_ and produced pigments even under repressing high nitrogen (60 mM glutamine) conditions, when neither bikaverin nor fusarubins are produced by the WT (**Figure 3A**). To show which of the pigments is de-regulated in the mutant, we studied the expression of bikaverin and fusarubin biosynthetic genes under the different nitrogen conditions (6 and 60 mM glutamine, and 6 mM NaNO_3_, respectively) in the WT, the *CSM1* deletion mutant and the complemented strain *CSM1*^C^ *^CSM*1*^* (**Figure 3B**). Both, the bikaverin and fusarubin biosynthetic genes were differently expressed compared to the WT. Bikaverin biosynthetic genes are usually strongly repressed under nitrogen sufficiency, but in Δ*csm1* they were highly expressed at 60 mM glutamine indicating that the nitrogen repression is overruled in the mutant (**Figure 3B**, shown for *BIK2* encoding a monooxygenase of the bikaverin gene cluster). Previous studies showed that fusarubin biosynthetic genes are only expressed after 4 days in the WT (Studt et al., 2012). In our study, we showed that *FSR2* (encoding an *O*-methyltransferase of the fusarubin gene cluster) was already expressed in Δ*csm1* after 2 days under inducing conditions (6 mM NaNO_3_, **Figure 3B**). Surprisingly, *FSR2* is also highly expressed in the Δ*csm1* strain under repressing acidic conditions (6 mM glutamine) in contrast to the WT and the complemented strain. Quantification of bikaverin and fusarubins yields in the supernatant of 7-day-old cultures by HPLC analysis confirmed the expression data (**Figure 3C**).

Taken together, the deletion of *CSM1* results in drastically increased formation of microconidia, higher sensitivity to oxidative stress and de-regulated biosynthesis of both red pigments.

### Is the Conserved RQSLPSI Motif Critical for Csm1 Function?

In contrast to the other known fungal GATA TFs, such as SreA, AreA, AreB, WC-1, and WC-2, all NsdD orthologs including Ltf1 from *B. cinerea* and Csm1 from *F. fujikuroi* contain the highly conserved RQSLPSI motif in the N-terminal regions (**Figure 1A**) that has not been studied before. To show whether this seven amino acid-motif is necessary for Csm1 function, we generated a construct containing a mutated gene copy embedded between the 5′ and 3′ flanks of *CSM1* leading to three amino acid exchanges in this domain (R57G; S59A; S62A) (*CSM1*^MUT^ Supplementary Figure S1). PCR fragments consisting of the flanks, a nourseothricin resistance cassette and the mutated gene fused/not fused to *GFP*, were introduced into the deletion mutant by homologous recombination. All transformants showed WT-like growth on three different agar media (Supplementary Figures S3A,B). Furthermore, the transformants carrying the mutated copy at the *CSM1* locus were studied for conidiation, and fusarubins production in comparison to the WT (Supplementary Figures S3C–E). In addition, we examined the subcellular localization of Csm1-gfp and Csm1^MUT^-gfp by fluorescence microscopy. The localization of the fusion proteins was determined after growth for 24 h in ICI medium supplemented with either 6 mM glutamine or 6 mM NaNO_3_. Both Csm1-gfp fusion proteins were always localized to the nucleus irrespective of the nitrogen source (Supplementary Figure S3F).

In summary, the strains carrying the mutated *CSM1* copy behave like the WT with respect to colony morphology, conidia formation, pigmentation, fusarubin gene expression, and subcellular localization indicating that the conserved motif does not affect the functionality of Csm1 (Supplementary Figure S3).

### Are Ltf1 from *B. cinerea* and Csm1 from *F. fujikuroi* Interchangeable?

Ltf1 from *B. cinerea* and Csm1 from *F. fujikuroi* are orthologous GATA TFs with an overall level of protein sequence similarity of about 30%, mainly due to the almost identical zinc finger DNA binding domains (**Figure 1A**). Ltf1 regulates growth and conidia formation similar to Csm1 in *F. fujikuroi*. However, Ltf1 has *Botrytis*-specific functions in maintaining ROS homoeostasis, especially during light exposure, virulence, and secondary metabolism (Schumacher et al., 2014). To establish whether both TFs can replace each other despite the low sequence similarity and species-specific functions, we performed a cross-species complementation experiment. *LTF1*, driven by the *CSM1* promoter, was introduced into the Δ*csm1* mutant by integration at the *CSM1* locus (*CSM1*^C^ *^LTF*1*^*, Supplementary Figure S1B). Surprisingly, all functions impaired in the Δ*csm1* mutant (growth, conidiation, and WT-like expression of the fusarubin biosynthetic genes) were fully restored by expressing *LTF1* in the Δ*csm1* background (**Figures 2A,B**, **3A,B**).

**FIGURE 4:**
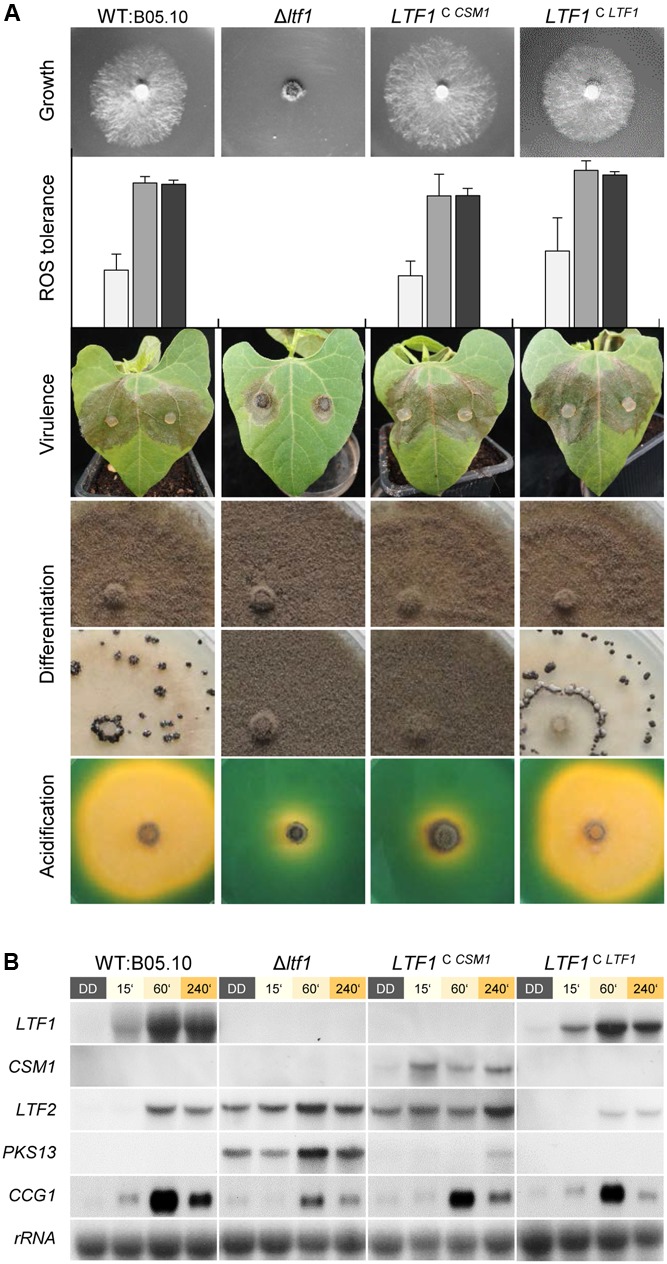
Csm1 is able to fulfill some, but not all functions of *B. cinerea* Ltf1. *CSM1* was integrated at the *LTF1* locus in Δ*ltf1*. The *LTF1*-complemented mutant (*LTF1*^C^ *^LTF*1*^*) was included as positive control (Schumacher et al., 2014). **(A)** Growth on minimal medium (CD) was monitored after 3 days of incubation in light/dark conditions. Reactive oxygen species (ROS) tolerance was tested using CM supplemented with 7.5 mM H_2_O_2._ Strains were cultivated for 3 days in constant light (white bars), light/dark (gray bars) or constant darkness (black bars). Shown are the mean values and standard deviations of colony diameters deriving from four colonies per strain and condition. Virulence was monitored on primary leaves of *P. vulgaris* which were inoculated with plugs of vegetative mycelia. Images were taken after 3 days of incubation. Differentiation phenotypes in light (upper row) and in darkness (lower row) were documented after 2 weeks of incubation on solid CM. Acidification of the culture medium was visualized by cultivating the strains on solid CM (pH 7.5) supplemented with the pH indicator bromothymol blue. Images were taken after 5 days of incubation. The color change from green to yellow indicates acidification (pH < 6). **(B)** Northern blot analysis demonstrates the light-responsive expression of *CSM1* and the de-regulation of *LTF2* in *CSM1*-expressing mutants. Dark-grown cultures (50 h) of the indicated strains were exposed to light for the indicated times, or kept in darkness for further 60 min before harvest.

A similar strategy was used to introduce *CSM1* at the *LTF1* locus in the *B. cinerea* Δ*ltf1* mutant that resulted in light-responsive expression of *CSM1* (*LTF1*^C^ *^CSM*1*^*, **Figure 4**). *CSM1* rescued the defects in growth on CD, ROS tolerance (growth on CM in presence of H_2_O_2_), and in virulence on bean plants, but failed to restore light-dependent differentiation (sclerotial development in constant darkness) and the capacity to acidify the culture medium (**Figure 4A**). The Ltf1-dependent TF *LTF2*, encoding the major, positive-acting regulator of conidiation in *B. cinerea* (Cohrs et al., 2016) was still expressed in the *LTF1*^C^ *^CSM*1*^* mutants in the dark, a result which is in accordance with the observed production of conidia under these otherwise repressing conditions. Nevertheless, expression of *PKS13* encoding the key enzyme for conidial melanogenesis (Schumacher, 2016), was significantly repressed (**Figure 4B**). Taken together, *CSM1* is able to fulfill some, but not all, functions of *LTF1* in *B. cinerea*.

### Csm1 Affects Transcription of a Large Set of Genes

Altered growth and conidia formation as well as significant changes in pigmentation suggested a global impact of this GATA TF on primary and secondary metabolism of the fungus. To gain deeper insight into the role Csm1 plays in *F. fujikuroi*, a genome-wide expression analysis was performed using Roche NimbleGen DNA microarrays that were manufactured and based on the present genome annotation of *F. fujikuroi* IMI 58289 (Wiemann et al., 2013). Total RNA was extracted from mycelia of the WT and Δ*csm1* mutant which were grown for 3 days under acidic low and high nitrogen (6 and 60 mM glutamine) and alkaline low nitrogen (12 mM NaNO_3_) conditions. Then, by use of the twofold change in expression at the 95% confidence interval as selection criterion we searched for differentially expressed genes. Altogether, 2309 genes were significantly up- or down-regulated in the mutant under at least one condition. From those genes, 49 and 27 are significantly up- and down-regulated, respectively, under all three conditions suggesting a nitrogen-independent control of these genes by Csm1. Under both nitrogen-limiting conditions (6 mM glutamine; 12 mM NaNO_3_), 170 and 129 genes were up- and down-regulated, respectively. Among these are several physically linked genes that may be involved in the same metabolic processes (functional clusters). Examples are the genes *FFUJ_11793* – *FFUJ_11797*, all of which are involved in glutamate, arginine and proline metabolism and all down-regulated in the mutant at low nitrogen (Supplementary Table S2A). Other examples of genes that belong to the most up-regulated genes in the mutant under low nitrogen are two adjacent genes (*FFUJ_10257* and *FFUJ_10258*) encoding a cytochrome b5 reductase and a P450 monooxygenase, and four genes (*FFUJ_10843* – *FFUJ_10846*) that are probably involved in the conversion of gamma-glutamylamines to free amines and 5-oxoproline. Functional classification of the Csm1 target genes revealed that 92 SM biosynthetic genes, 178 transporter-encoding genes, 108 TFs, 88 dehydrogenases, 70 P450 and FAD-dependent monooxygenases, 14 histone modifying genes, and 11 putative ROS-detoxifying genes were affected by deletion of *CSM1* (Supplementary Tables S2C–H). The differential expression of two randomly selected transporter-encoding genes was confirmed by qRT-PCR (Supplementary Figure S4A).

A functional distribution analysis of the full up- or down-regulated gene sets for all three conditions indicates a strong enrichment of genes annotated in the categories ‘secondary metabolism,’ ‘disease, virulence, and defense,’ ‘transport facilities,’ and other categories, confirming a general deregulation of these processes in the *CSM1* deletion (Supplementary Table S3).

At least one potential Csm1-binding site ([G/T][C/G] GATAA) as shown for NsdD in *A. nidulans* (Lee et al., 2016) was found in the promoters of a subset of the genes affected in the Δ*csm1* mutant (Supplementary Table S2), but also in promoters which are not significantly up- or down-regulated in our data sets. Therefore, the *A. nidulans* motif seems most likely not to be identical for Csm1 in *F. fujikuroi* and we suggest that the presence of these motifs does not mean that they are functional.

### Csm1 is a Global Regulator of Secondary Metabolism

Of the 47 putative gene clusters in the genome of *F. fujikuroi*, 19 were affected in the mutant under at least one condition. Among them are the genes involved in biosynthesis of apicidin F, beauvericin, bikaverin, fusarubins, fusarin C, fusaric acid, fujikurins, gibepyrones, and fumonisins (**Table 1**). The data for some of the key enzyme-encoding genes that were found to be differentially expressed in the microarray were validated by qRT-PCR (Supplementary Figure S4B).

**Table 1:** Differentially expressed secondary metabolite (SM) gene clusters in wild-type (WT) and Δ*csm1*.

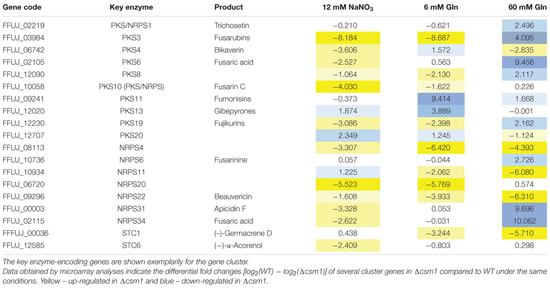

For some of the known gene clusters, we observed not only an up- or down-regulation under the inducing production conditions, but also a de-regulation regarding nitrogen and pH responses. For instance, the genes for biosynthesis of apicidin F, fusaric acid, fusarin C, and beauvericin were up-regulated under usually repressing low nitrogen conditions, either at low nitrate (alkaline pH) or at low glutamine (acidic pH) (**Table 1** and Supplementary Table S2B). The microarray data also confirmed the elevated and de-regulated expression of bikaverin and fusarubin genes shown by northern blot analysis (**Figure 3**). The PKS-derived red pigment bikaverin is produced under normally repressing high nitrogen (60 mM glutamine) and low nitrogen alkaline (6 mM NaNO_3_) conditions in the Δ*csm1* strain indicating the decoupling of its biosynthesis from both nitrogen repression and PacC-mediated pH regulation (**Figure 3B**). The most prominent de-regulation was observed for the fusarubin gene cluster that is highly expressed under acidic conditions (6 mM glutamine) in contrast to the WT.

Among the differentially regulated gene clusters are also six uncharacterized clusters: three NRPS (NRPS4, NRPS11, and NRPS20), two PKS (PKS8 and PKS20), and one sesquiterpene cluster (STC1) (**Table 1**). The three NRPS- and the STC1-encoding genes are highly up-regulated in the mutant. Therefore, the deletion of the GATA TF Csm1 is a useful tool to identify new SMs by activating the biosynthetic genes clusters and to link them with the respective biosynthetic gene cluster.

### Identification of the STC1 Product Using the Δ*csm1* Mutant

**FIGURE 5:**
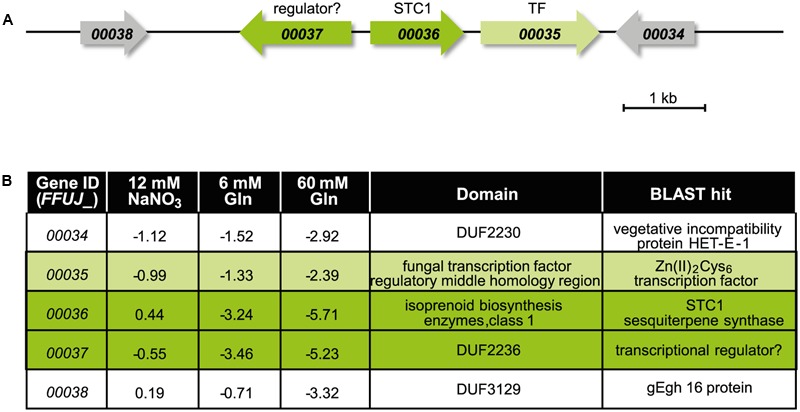
The putative STC1 cluster is up-regulated in the Δ*csm1* mutant. **(A)** Cluster organization of the putative STC1 gene cluster. Arrows indicate the direction of transcription. **(B)** Expression levels of *STC1* (*FFUJ_00036*) and adjacent genes in the Δ*csm1* mutant compared to the WT in the microarray analysis. Protein domains were predicted by BlastP search.

In our study we took a closer look to the putative STC1 cluster because *STC1* (*FFUJ_00036*) and one adjacent gene with unknown function (*FFUJ_00037*) are up-regulated in the Δ*csm1* strain in media with both 6 and 60 mM glutamine (**Figure 5** and Supplementary Table S2). The genes upstream (*FFUJ_00034* and *FFUJ_00035*) encoding a vegetative incompatibility protein HET-E-1 and a Zn(II)_2_Cys_6_ TF, respectively, and one gene downstream (*FFUJ_00038*) were only slightly up-regulated at 60 mM glutamine (**Figure 5B**).

To identify the product of STC1, the protein was heterologously expressed in *E. coli*. Reverse transcription of the mRNA from the Δ*csm1* and amplification of the cDNA by PCR allowed gene cloning into the pYE-Express shuttle vector (Dickschat et al., 2014) to yield the expression construct pYE-Express-*STC1*. The protein was purified and incubated with geranyl (GPP), farnesyl (FPP), and geranylgeranyl (GGPP) diphosphate, the substrates for mono-, sesqui- and diterpene cyclases, respectively. Subsequent GC/MS analysis revealed that a sesquiterpene hydrocarbon ([M]^+^: *m*/*z* = 204.35) was formed only from FPP, whereas GPP and GGPP were not converted. The sesquiterpene hydrocarbon was identified as germacrene D by GC/MS. This compound is known as a major constituent present in the essential oil of higher plants (van Der Hoeven et al., 2000; Aoki et al., 2010).

**FIGURE 6:**
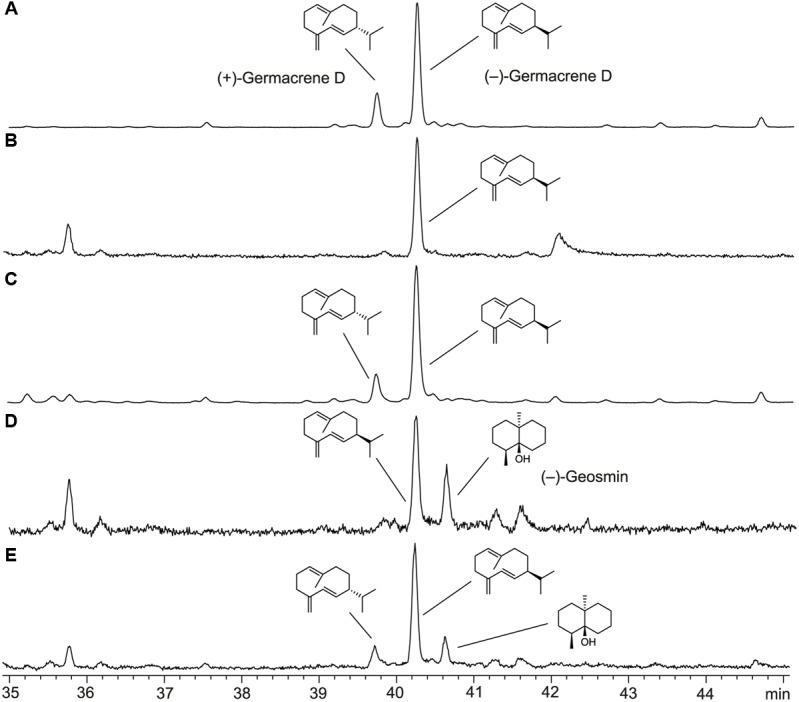
Total ion chromatograms of GC-MS analyses using a homochiral stationary phase. **(A)** Essential oil of *Solidago canadensis*, **(B)** product of STC1 from *F. fujikuroi*, **(C)** co-injection of **A,B**, **(D)** products obtained from FPP with geosmin synthase from *S. flavochromogenes*, **(E)** co-injection of **A,D**.

The absolute configuration of the STC1 product, germacrene D, was determined by GC analysis on a homochiral stationary phase. The essential oil of *Solidago canadensis*, a herbaceous perennial of the family *Asteraceae*, contains a mixture of both enantiomers of germacrene D in the leaves (Schmidt et al., 1998). Separation of the two enantiomers could be achieved (**Figure 6**), but since the enantiomeric ratio of germacrene D varies in different *Solidago* species (Niwa et al., 1980), an independent sample of one of the pure enantiomers was required for an unambiguous peak assignment. The bacterial (–)-geosmin synthase is known to make the side product (–)-germacrene D (Jiang et al., 2006). To obtain this compound as reference material, the (–)-geosmin synthase gene from *Streptomyces flavochromogenes* (NCBI accession number: WP_030314776) was cloned into pYE-Express and the purified protein was used to convert FPP into a mixture of (–)-germacrene D and (–)-geosmin. Comparison to the STC1 product identified the sesquiterpene from *F. fujikuroi* unambiguously as (–)-germacrene D.

## Discussion

In this study, we characterized the first NsdD ortholog in a *Fusarium* species. NsdD and its orthologs are one of the six characterized GATA factors in fungi beside the nitrogen regulators AreA and AreB, the central components of the blue light-sensing system WC-1 and WC-2, and the iron uptake regulator SreA (Machida and Gomi, 2010). NsdD has been identified as activator of sexual and repressor of asexual development in *A. nidulans* (Han et al., 2001; Lee et al., 2014). NsdD orthologs in *A. flavus* and *A. fumigatus* play similar roles as activators of sexual development and repressors of conidiation (Grosse and Krappmann, 2008; Szewczyk and Krappmann, 2010). NsdD orthologs were also shown to affect the production of SMs such as a dark mycelial pigment and gliotoxin in *Aspergillus* spp. (Szewczyk and Krappmann, 2010; Lee et al., 2016) and several SM gene clusters, e.g., those for botcinic acid and botrydial biosynthesis, in *B. cinerea* (Schumacher et al., 2014). In addition, Sub-1 in *N. crassa* (Colot et al., 2006; Chen et al., 2009) and Ltf1 in *B. cinerea* (Schumacher et al., 2014) act as light-responsive TFs controlling a whole set of light-regulated genes, while Δ*pro44* mutants in *S. macrospora* (Nowrousian et al., 2012) show comparable vegetative growth in light and darkness and are probably not involved in light signaling.

### Csm1 in *F. fujikuroi* Has Common and Different Functions Compared to Its Orthologs

Deletion of *CSM1* resulted in a slight growth reduction on solid media, as described for the deletion of the orthologous genes. However, the Δ*csm1* mutant of *F. fujikuroi* has no growth defect in constant light. This is in marked contrast to the *B. cinerea* Δ*ltf1* mutant that is incapable of growth in light, most likely due to the specific role of Ltf1 to maintain the ROS homoeostasis (Schumacher et al., 2014). Nevertheless, the observations that the Δ*csm1* mutant displays an increased sensitivity to H_2_O_2_, especially on synthetic minimal agar (CD) in the light, and that several catalases, peroxidases, and heme peroxidases were up-regulated, suggest a role of Csm1 in maintaining the ROS balance (Supplementary Table S2). Furthermore, the mutant accumulates more orange pigmentation (most likely carotenoids) on CM agar in light and in darkness pointing out the bypass of light for induction of carotenoid biosynthesis.

In a similar manner to the other NsdD orthologs, Csm1 acts as a strong repressor of conidia formation, both under inducing light/dark and neutral dark conditions. The Δ*csm1* mutant produces about 10-fold more microconidia than the WT. Previously, we have shown that the *F. fujikuroi* Δ*vel1* mutant lost the ability to generate microconidia (Wiemann et al., 2010). However, deletion of *CSM1* in the Δ*vel1* background partially restored microconidial formation underlining the role of Csm1 as a major negative-acting regulator of conidiation in *F. fujikuroi*.

Moreover, analysis of *A. fumigatus* Δ*nsdD* mutants suggests a role of this conserved regulator in cell wall stress resistance and hyphal fusion accompanying heterokaryon formation (Szewczyk and Krappmann, 2010). Furthermore, the absence of sclerotia in *A. flavus* Δ*nsdD* mutants may be due to defects in hyphal fusion suggesting that NsdD may regulate genes required for formation of hyphal anastomosis as well as cell wall integrity and maintenance (Cary et al., 2012). The microarray data in the present study revealed the up- and down-regulation of about 20 heterokaryon incompatibility (HET) genes in the mutant. For instance, there are two adjacent HET genes, *FFUJ_06507* and *FFUJ_06508*, that were down-regulated in the mutant under all three conditions used (Supplementary Table S2).

### Cross-Species Complementation

To establish whether Csm1 and Ltf1 from *F. fujikuroi* and *B. cinerea*, respectively, are able to take over common and/or different functions in the other fungus, we performed cross-species complementation. Surprisingly, Ltf1 from *B. cinerea* restored not only WT-like conidiation, a function that have both GATA factors in common, but also the *F. fujikuroi*-specific fusarubins production, growth behavior and colony morphology. In contrast, Csm1 from *F. fujikuroi* fully restored only virulence on French bean, WT-like growth on minimal medium and H_2_O_2_ tolerance. However, the *B. cinerea*-specific light-dependent differentiation program (conidiation in light, sclerotia formation in the dark) (Schumacher et al., 2014) is not restored by the *Fusarium* ortholog, suggesting that the repression of conidiation in *B. cinerea* by Ltf1 requires specific co-regulators that fail to interact with *F. fujikuroi* Csm1.

A similar partial restoration of the WT phenotype has been observed by heterologous expression of *Penicillium chrysogenum VelA* and *LaeA* in the *F. fujikuroi* Δ*vel1* and Δ*lae1* mutants, respectively. Although growth and conidiation were rescued, the biosynthesis of the *F. fujikuroi-*specific gibberellins were not restored by neither PcVelA nor PcLaeA (Wiemann et al., 2010). In contrast, full restoration of the WT phenotype, including penicillin production, was achieved by heterologous expression of *F. fujikuroi VEL1* and *LAE1* in the respective *P. chrysogenum* mutants (Hoff et al., 2010).

### Secondary Metabolism Is Influenced by Csm1

One of the most obvious phenotypes of the Δ*csm1* mutant is the deep red pigmentation of liquid cultures, under inducing and repressing conditions for bikaverin and fusarubins biosynthesis. Northern blot analysis revealed a strong up-regulation of bikaverin genes under repressing nitrogen-sufficient conditions, and an earlier and de-regulated expression of the fusarubins genes under optimal low nitrogen alkaline and repressing acidic conditions, respectively. For bikaverin biosynthetic genes, a similar de-regulation has been observed in the Δ*vel1* mutant where the expression of these genes was still detected under high nitrogen (60 mM glutamine) and even alkaline (6 and 120 mM NaNO_3_) conditions in contrast to the WT (Wiemann et al., 2010).

The altered pigmentation of the mutant cultures indicated that Csm1 plays a role in regulation of secondary metabolism besides affecting growth and differentiation. Microarray analysis revealed that 19 of the 47 SM gene clusters were affected in their gene expression. Beside the two red pigments, several clusters with known products are also up- or down-regulated. The otherwise silent beauvericin gene cluster that has been recently shown to be activated by histone modifications (Niehaus et al., 2016b) was strongly up-regulated in low and high glutamine conditions, and the fusarin C biosynthetic genes are highly expressed under repressing low nitrate conditions. The fusaric acid and fumonisin gene clusters are repressed in the mutant under inducing high and low nitrogen conditions, respectively. Among the affected gene clusters are also six with yet unknown products. The most up-regulated cryptic cluster genes are *STC1*, *NRPS20*, *NRPS4*, and *NRPS11*. These results indicate that genome-wide expression analyses of WT and regulator mutants are powerful approaches for identifying unknown SMs and to subsequently link them to their biosynthetic genes.

A role in regulating SM genes has been demonstrated also for NsdD orthologs in other fungi. In *A. flavus*, NsdD affects morphogenesis and aflatoxin biosynthesis (Cary et al., 2012). Deletion of *LTF1* in *B. cinerea* affects the expression of known (e.g., for botcinic acid, botrydial and DHN melanin) and yet uncharacterized SM biosynthetic genes (Schumacher et al., 2014; Viaud et al., 2016). These data support the general observation that major regulators of differentiation, such as orthologs of *A. nidulans* VeA, LaeA, MtfA, and NsdD are also regulators of secondary metabolism (Bayram and Braus, 2012; Cary et al., 2015; Lind et al., 2015, 2016; Schumacher et al., 2015; Zhuang et al., 2016).

### Identification of the STC1 Product

One of the most up-regulated SM key genes is *STC1* encoding one of the nine sesquiterpene cyclases (STC1-STC9) in the *F. fujikuroi* genome (Niehaus et al., 2016a). The gene next to *STC1* (*FFUJ_00037*) is transcribed from the same promoter in opposite direction and displays similarly elevated expression levels. Such a bidirectional arrangement of two co-regulated genes has also been reported for several fungal SM gene clusters in *F. fujikuroi*. Examples are *CPS/KS* and *GGS2* (encoding an *ent*-copalyl/*ent*-kaurene synthase and a geranylgeranyl diphosphate synthase, respectively), the key enzyme-encoding genes of gibberellic acid biosynthesis (Bömke and Tudzynski, 2009), or *BEA1* and *BEA2* (encoding a NRPS and a 2-ketoisovalerate reductase, respectively) as biosynthetic genes of the recently identified beauvericin cluster (Niehaus et al., 2016b). For *BEA1*/*BEA2* it was shown that a shared sequence element for TF binding allows coordinated expression of both genes (Niehaus et al., 2016b). In contrast to the two putative STC1 cluster genes (*FFUJ_00036* and *FFUJ_00037*), the three remaining potential cluster genes (*FFUJ_00034*, *FFUJ_00035*, and *FFUJ_00038*) are up-regulated in the Δ*csm1* mutant but to lesser extents.

Previously, we succeeded in functionally characterizing of four STC-encoding genes in *F. fujikuroi*. These studies revealed that STC6 is an (–)-α-acorenol synthase and STC4 is a (+)-koraiol synthase (Brock et al., 2011, 2013). Both sesquiterpene alcohols are the main constituents of *F. fujikuroi* headspace extracts. Recently, the products of STC3 and STC5 were identified by heterologous protein expression in *E. coli*, followed by enzyme purification and incubations with FPP as (+)-eremophilene and (–)-guaia-6,10(14)-diene, respectively (Burkhardt et al., 2016). A similar approach was used in this study to identify the STC1 product as (–)-germacrene D that was unambiguously identified by comparison via GC/MS on a homochiral stationary phase to authentic standards in the essential oil from *Solidago canadensis* and obtained from FPP with the geosmin synthase from *Streptomyces flavochromogenes*. Germacrene D is known to be produced by a number of plants and has antimicrobial and insecticidal properties (van Der Hoeven et al., 2000; Aoki et al., 2010). Due to these biological activities, and because of the fact that the chemical synthesis of sesquiterpenes is a highly complex and time-consuming approach, the biosynthesis of those compounds by genetically engineered fungal mutants or by heterologous expression of the respective terpene cyclases is of biotechnological interest.

A phylogenetic analysis of 2500 terpene cyclase homologs reveals that the cyclases for (–)-germacrene D from plants and fungi have evolved independently (Supplementary Figure S5). Furthermore, terpene cyclases with the structurally and biosynthetically closely related product (–)-germacrene D-4-ol are encoded in a number of bacteria (Rinkel et al., 2016), but also these enzymes are phylogenetically distant to the fungal (–)-germacrene D synthases.

The (–)-germacrene D synthase-encoding gene (*STC1*) is embedded in a cluster of four additional potential cluster genes. The Zn(II)_2_Cys_6_ TF-encoding gene (*FFUJ_00035*) and a gene with a DUF2236 domain (*FFUJ_00037*) could be involved in regulation of the cluster genes. Protein BLAST search revealed 80 and 64% identity of the DUF2236 domain-containing protein with a TF in *Fusarium langsethiae* (KPA40612.1) and a transcriptional regulator in *Alternaria alternata* (XP_018391512.1), respectively. The function of the remaining putative cluster genes (*FFUJ_00034* and *FFUJ_00038*) and the structural elucidation of the final product after potential modification of (–)-germacrene D by these cluster-encoded enzymes are currently being studied (Arndt et al., unpublished).

## Author Contributions

BT, E-MN, JS, and JD contributed to the design of the work. E-MN, IB, and JS were involved in data acquisition. E-MN, JD, JS, MM, and UG were involved in data analysis. BT, E-MN, JS, and JD wrote the manuscript. All authors revised and approved the manuscript.

## Conflict of Interest Statement

The authors declare that the research was conducted in the absence of any commercial or financial relationships that could be construed as a potential conflict of interest.

## Footnotes

1 http://www.ncbi.nlm.nih.gov/geo/query/acc.cgi?acc=GSE90945

## References

[B1] Aoki K. Yano K. Suzuki A. Kawamura S. Sakurai N. Suda K. (2010). Large-scale analysis of full-length cDNAs from the tomato (*Solanum lycopersicum*) cultivar Micro-Tom, a reference system for the Solanaceae genomics. *BMC Genomics* 11:210 10.1186/1471-2164-11-210 PMC2859864 20350329

[B2] Bayram O. Krappmann S. Ni M. Bok J. W. Helmstaedt K. Valerius O. (2008). VelB/VeA/LaeA complex coordinates light signal with fungal development and secondary metabolism. *Science* 320 1504–1506. 10.1126/science.1155888 18556559

[B3] Bayram Ö. Braus G. H. (2012). Coordination of secondary metabolism and development in fungi: the velvet family of regulatory proteins. *FEMS Microbiol. Rev.* 36 1–24. 10.1111/j.1574-6976.2011.00285.x 21658084

[B4] Bok J. W. Keller N. P. (2004). LaeA, a regulator of secondary metabolism in *Aspergillus* spp. *Eukaryot. Cell* 3 527–535. 10.1128/EC.3.2.527-535.2004 15075281 PMC387652

[B5] Bömke C. Tudzynski B. (2009). Diversity, regulation, and evolution of the gibberellin biosynthetic pathway in fungi compared to plants and bacteria. *Phytochemistry* 70 1876–1893. 10.1016/j.phytochem.2009.05.020 19560174

[B6] Brakhage A. A. (2013). Regulation of fungal secondary metabolism. *Nature Rev. Microbiol.* 11 21–32. 10.1038/nrmicro2916 23178386

[B7] Brock N. L. Huss K. Tudzynski B. Dickschat J. S. (2013). Genetic dissection of sesquiterpene biosynthesis by *Fusarium fujikuroi*. *ChemBioChem* 14 311–315. 10.1002/cbic.201200695 23335243

[B8] Brock N. L. Tudzynski B. Dickschat J. S. (2011). Biosynthesis of sesqui-and diterpenes by the gibberellin producer *Fusarium fujikuroi*. *ChemBioChem* 12 2667–2676. 10.1002/cbic.201100516 21990128

[B9] Burkhardt I. Siemon T. Henrot M. Studt L. Rösler S. Tudzynski B. (2016). Mechanistic characterisation of two sesquiterpene cyclases from the plant pathogenic fungus *Fusarium fujikuroi*. *Angew. Chem. Int. Ed. Engl.* 55 8748–8751. 10.1002/anie.201603782 27294564

[B10] Büttner P. Koch F. Voigt K. Quidde T. Risch S. Blaich R. (1994). Variations in ploidy among isolates of *Botrytis cinerea*: implications for genetic and molecular analyses. *Curr. Genet.* 25 445–450. 10.1007/BF00351784 8082191

[B11] Calvo A. M. Wilson R. A. Bok J. W. Keller N. P. (2002). Relationship between secondary metabolism and fungal development. *Microbiol. Mol. Biol. Rev.* 66 447–459. 10.1128/MMBR.66.3.447-459.2002 12208999 PMC120793

[B12] Canessa P. Schumacher J. Hevia M. A. Tudzynski P. Larrondo L. F. (2013). Assessing the effects of light on differentiation and virulence of the plant pathogen *Botrytis cinerea*: characterization of the White Collar Complex. *PLoS ONE* 8:e84223 10.1371/journal.pone.0084223 PMC3877267 24391918

[B13] Cary J. W. Han Z. Yin Y. Lohmar J. M. Shantappa S. Harris-Coward P. Y. (2015). Transcriptome analysis of *Aspergillus flavus* reveals veA-dependent regulation of secondary metabolite gene clusters, including the novel aflavarin cluster. *Eukaryot. Cell* 14 983–997. 10.1128/EC.00092-15 26209694 PMC4588625

[B14] Cary J. W. Harris-Coward P. Y. Ehrlich K. C. Mack B. M. Kale S. P. Larey C. (2012). NsdC and NsdD affect *Aspergillus flavus* morphogenesis and aflatoxin production. *Eukaryot. Cell* 11 1104–1111. 10.1128/EC.00069-12 22798394 PMC3445981

[B15] Cenis J. L. (1992). Rapid extraction of fungal DNA for PCR amplification. *Nucleic Acids Res.* 20:2380 10.1093/nar/20.9.2380 PMC312363 1594460

[B16] Chen C. H. Ringelberg C. S. Gross R. H. Dunlap J. C. Loros J. J. (2009). Genome-wide analysis of light-inducible responses reveals hierarchical light signalling in *Neurospora*. *EMBO J.* 28 1029–1042. 10.1038/emboj.2009.54 19262566 PMC2683703

[B17] Cohrs K. C. Simon A. Viaud M. Schumacher J. (2016). Light governs asexual differentiation in the grey mould fungus *Botrytis cinerea* via the putative transcription factor BcLTF2. *Environ. Microbiol.* 18 4068–4086. 10.1111/1462-2920.13431 27347834

[B18] Colot H. V. Park G. Turner G. E. Ringelberg C. Crew C. M. Litvinkova L. (2006). A high-throughput gene knockout procedure for *Neurospora* reveals functions for multiple transcription factors. *Proc. Natl. Acad. Sci. U.S.A.* 103 10352–10357. 10.1073/pnas.0601456103 16801547 PMC1482798

[B19] Darken M. A. Jensen A. L. Shu P. (1959). Production of gibberellic acid by fermentation. *Appl. Microbiol.* 7 301–303. 13814121 10.1128/am.7.5.301-303.1959 PMC1057525

[B20] Dickschat J. S. Pahirulzaman K. A. Rabe P. Klapschinski T. A. (2014). An improved technique for the rapid chemical characterisation of bacterial terpene cyclases. *ChemBioChem* 15 810–814. 10.1002/cbic.201300763 24573945

[B21] Dunlap J. C. (1999). Molecular bases for circadian clocks. *Cell* 96 271–290. 10.1016/S0092-8674(00)80566-8 9988221

[B22] Estrada A. F. Avalos J. (2008). The White Collar protein WcoA of *Fusarium fujikuroi* is not essential for photocarotenogenesis, but is involved in the regulation of secondary metabolism and conidiation. *Fungal Genet. Biol.* 45 705–718. 10.1016/j.fgb.2007.12.003 18203635

[B23] Fisher R. A. (1922). On the interpretation of χ 2 from contingency tables, and the calculation of P. *J. R. Stat. Soc.* 85 87–94.

[B24] Geissman T. A. Verbiscar A. J. Phinney B. O. Cragg G. (1966). Studies on the biosynthesis of gibberellins from *ent*-kaurenoic acid in cultures of *Gibberella fujikuroi*. *Phytochemistry* 5 933–947. 10.1016/S0031-9422(00)82790-9

[B25] Grosse V. Krappmann S. (2008). The asexual pathogen *Aspergillus fumigatus* expresses functional determinants of *Aspergillus nidulans* sexual development. *Eukaryot. Cell* 7 1724–1732. 10.1128/EC.00157-08 18757566 PMC2568067

[B26] Han K. Han K. Yu J. Chae K. Jahng K. Han D. (2001). The *nsdD* gene encodes a putative GATA-type transcription factor necessary for sexual development of *Aspergillus nidulans*. *Mol. Microbiol.* 41 299–309. 10.1046/j.1365-2958.2001.02472.x 11489119

[B27] Hoff B. Kamerewerd J. Sigl C. Mitterbauer R. Zadra I. Kurnsteiner H. (2010). Two components of a velvet-like complex control hyphal morphogenesis, conidiophore development, and penicillin biosynthesis in *Penicillium chrysogenum*. *Eukaryot. Cell* 9 1236–1250. 10.1128/EC.00077-10 20543063 PMC2918941

[B28] Janevska S. Arndt B. Niehaus E.-M. Burkhardt I. Rösler S. M. Brock N. L. (2016). Gibepyrone biosynthesis in the rice pathogen *Fusarium fujikuroi* is facilitated by a small polyketide synthase gene cluster. *J. Biol. Chem.* 291 27403–27420. 10.1074/jbc.M116.753053 27856636 PMC5207165

[B29] Jiang J. He X. Cane D. E. (2006). Geosmin biosynthesis. *Streptomyces coelicolor* germacradienol/germacrene D synthase converts farnesyl diphosphate to geosmin. *J. Am. Chem. Soc.* 128 8128–8129. 10.1021/ja062669x 16787064

[B30] Kangatharalingam N. Ferguson M. W. (1984). A simple and rapid technique for fluorescence staining of fungal nuclei. *Curr. Microbiol.* 10 99–103. 10.1007/BF01575767

[B31] Kato N. Brooks W. Calvo A. M. (2003). The expression of sterigmatocystin and penicillin genes in *Aspergillus nidulans* is controlled by veA, a gene required for sexual development. *Eukaryot. Cell* 2 1178–1186. 10.1128/EC.2.6.1178-1186.2003 14665453 PMC326641

[B32] Keller N. P. Nesbitt C. Sarr B. Phillips T. D. Burow G. B. (1997). pH regulation of sterigmatocystin and aflatoxin biosynthesis in *Aspergillus* spp. *Phytopathology* 87 643–648. 10.1094/PHYTO.1997.87.6.643 18945083

[B33] Lee M. K. Kwon N. J. Choi J. M. Lee I. S. Jung S. Yu J. H. (2014). NsdD is a key repressor of asexual development in *Aspergillus nidulans*. *Genetics* 197 159–173. 10.1534/genetics.114.161430 24532783 PMC4012476

[B34] Lee M. K. Kwon N. J. Lee I. S. Jung S. Kim S. C. Yu J. H. (2016). Negative regulation and developmental competence in *Aspergillus*. *Sci. Rep.* 6:28874 10.1038/srep28874 PMC4929475 27364479

[B35] Lind A. L. Smith T. D. Saterlee T. Calvo A. M. Rokas A. (2016). Regulation of secondary metabolism by the velvet complex is temperature- responsive in *Aspergillus*. *G*3 6 4023–4033. 10.1534/g3.116.033084 PMC5144971 27694115

[B36] Lind A. L. Wisecaver J. H. Smith T. D. Feng X. Calvo A. M. Rokas A. (2015). Examining the evolution of the regulatory circuit controlling secondary metabolism and development in the fungal genus *Aspergillus*. *PLoS Genet.* 11:e1005096 10.1371/journal.pgen.1005096 PMC4364702 25786130

[B37] Machida M. Gomi K. (eds). (2010). *Aspergillus: Molecular Biology and Genomics*. Wymondham: Horizon Scientific Press.

[B38] Michielse C. Pfannmüller A. Macios M. Rengers P. Dzikowska A. Tudzynski B. (2014). The interplay between the GATA transcription factors AreA, the global nitrogen regulator and AreB in *Fusarium fujikuroi*. *Mol. Microbiol.* 91 472–493. 10.1111/mmi.12472 24286256

[B39] Mihlan M. Homann V. Liu T. D. Tudzynski B. (2003). AreA directly mediates nitrogen regulation of gibberellin biosynthesis in *Gibberella fujikuroi*, but its activity is not affected by NMR. *Mol. Microbiol.* 47 975–991. 10.1046/j.1365-2958.2003.03326.x 12581353

[B40] Niehaus E.-M. Janevska S. von Bargen K. W. Sieber C. M. K. Harrer H. Humpf H.-U. (2014). Apicidin F: characterization and genetic manipulation of a new secondary metabolite gene cluster in the rice pathogen *Fusarium fujikuroi*. *PLoS ONE* 9:e103336 10.1371/journal.pone.0103336 PMC4109984 25058475

[B41] Niehaus E.-M. Münsterkotter M. Proctor R. H. Brown D. W. Sharon A. Idan Y. (2016a). Comparative “omics” of the *Fusarium fujikuroi* species complex highlights differences in genetic potential and metabolite synthesis. *Genome Biol. Evol.* 8 3574–3599. 10.1093/gbe/evw259 28040774 PMC5203792

[B42] Niehaus E.-M. Studt L. von Bargen K. W. Kummer W. Humpf H. Reuter G. (2016b). Sound of silence: the beauvericin cluster in *Fusarium fujikuroi* is controlled by cluster-specific and global regulators mediated by H3K27 modification. *Environ. Microbiol.* 18 4282–4302. 10.1111/1462-2920.13576 27750383

[B43] Niwa M. Iguchi M. Yamamura S. (1980). Co-occurrence of (-) and (+)-germacrene-D in *Solidago altissima* L.: determination of the optical rotation of optically pure germacrene-D. *Chem. Pharm. Bull.* 28 997–999. 10.1248/cpb.28.997

[B44] Nowrousian M. Teichert I. Masloff S. Kück U. (2012). Whole-genome sequencing of *Sordaria macrospora* mutants identifies developmental genes. *G*3 2 261–270. 10.1534/g3.111.001479 PMC3284333 22384404

[B45] Pfannmüller A. Leufken J. Studt L. Michielse C. B. Sieber C. M. K. Güldener U. (2017). Comparative transcriptome and proteome analysis reveals a global impact of the nitrogen regulators AreA and AreB on secondary metabolism in *Fusarium fujikuroi*. *PLoS ONE* 12:e0176194 10.1371/journal.pone.0176194 PMC5404775 28441411

[B46] Pontecorvo G. Roper J. A. Chemmons L. M. Macdonald K. D. Bufton A. W. J. (1953). The genetics of *Aspergillus nidulans*. *Adv. Genet.* 5 141–238. 10.1016/s0065-2660(08)60408-3 13040135

[B47] Rinkel J. Rabe P. Garbeva P. Dickschat J. S. (2016). Lessons from 1,3-hydride shifts in sesquiterpene cyclizations. *Angew. Chem. Int. Ed.* 55 13593–13596. 10.1002/anie.201608042 27666571

[B48] Rösler S. M. Sieber C. M. K. Humpf H.-U. Tudzynski B. (2016). Interplay between pathway-specific and global regulation of the fumonisin gene cluster in the rice pathogen *Fusarium fujikuroi*. *Appl. Microbiol. Biotechnol.* 100 5869–5882. 10.1007/s00253-016-7426-7 26966024

[B49] Ruepp A. Zollner A. Maier D. Albermann K. Hani J. Mokrejs M. (2004). The FunCat, a functional annotation scheme for systematic classification of proteins from whole genomes. *Nucleic Acids Res.* 32 5539–5545. 10.1093/nar/gkh894 15486203 PMC524302

[B50] Sambrook J. Fritsch E. F. Maniatis T. (1989). *Molecular Cloning: A Laboratory Manual*, Vol. 2 Cold Spring Harbor, NY: Cold Spring Harbor Laboratory Press

[B51] Schmidt C. O. Bouwmeester H. J. de Kraker J. König W. A. (1998). Biosynthesis of (+)-and (-)-germacrene D in *Solidago canadensis*: isolation and characterization of two enantioselective germacrene D synthases. *Angew. Chem. Int. Ed.* 37 1400–1402. 10.1002/(SICI)1521-3773(19980605)37:10<1400::AID-ANIE1400>3.0.CO;2-I 29710884

[B52] Schumacher J. (2012). Tools for *Botrytis cinerea*: new expression vectors make the gray mold fungus more accessible to cell biology approaches. *Fung. Genet. Biol.* 49 483–497. 10.1016/j.fgb.2012.03.005 22503771

[B53] Schumacher J. (2016). DHN melanin biosynthesis in the plant pathogenic fungus *Botrytis cinerea* is based on two developmentally regulated key enzyme (PKS)-encoding genes. *Mol. Microbiol.* 99 729–748. 10.1111/mmi.13262 26514268

[B54] Schumacher J. Simon A. Cohrs K. C. Traeger S. Porquier A. Dalmais B. (2015). The VELVET complex in the gray mold fungus *Botrytis cinerea*: impact of *BcLAE1* on differentiation, secondary metabolism, and virulence. *Mol. Plant Microbe Interact.* 28 659–674. 10.1094/MPMI-12-14-0411-R 25625818

[B55] Schumacher J. Simon A. Cohrs K. C. Viaud M. Tudzynski P. (2014). The transcription factor BcLTF1 regulates virulence and light responses in the necrotrophic plant pathogen *Botrytis cinerea*. *PLoS Genet.* 10:e1004040 10.1371/journal.pgen.1004040 PMC3886904 24415947

[B56] Smith C. W. Dilday R. H. (2003). *Rice: Origin, History, Technology, and Production*. Hoboken, NJ: John Wiley & Sons.

[B57] Studt L. Janevska S. Niehaus E.-M. Burkhardt I. Arndt B. Sieber C. M. K. (2016). Two separate key enzymes and two pathway-specific transcription factors are involved in fusaric acid biosynthesis in *Fusarium fujikuroi*. *Environ. Microbiol.* 18 936–956. 10.1111/1462-2920.13150 26662839

[B58] Studt L. Wiemann P. Kleigrewe K. Humpf H.-U. Tudzynski B. (2012). Biosynthesis of fusarubins accounts for pigmentation of *Fusarium fujikuroi* perithecia. *Appl. Environ. Microbiol.* 78 4468–4480. 10.1128/AEM.00823-12 22492438 PMC3370568

[B59] Szewczyk E. Krappmann S. (2010). Conserved regulators of mating are essential for *Aspergillus fumigatus* cleistothecium formation. *Eukaryot. Cell* 9 774–783. 10.1128/EC.00375-09 20348388 PMC2863953

[B60] Tudzynski B. Homann V. Feng B. Marzluf G. (1999). Isolation, characterization and disruption of the *areA* nitrogen regulatory gene of *Gibberella fujikuroi*. *Mol. Gen. Genet.* 261 106–114. 10.1007/s004380050947 10071216

[B61] van Der Hoeven R. S. Monforte A. J. Breeden D. Tanksley S. D. Steffens J. C. (2000). Genetic control and evolution of sesquiterpene biosynthesis in *Lycopersicon esculentum* and *L. hirsutum*. *Plant Cell* 12 2283–2294. 10.1105/tpc.12.11.2283 11090225 PMC150174

[B62] Viaud M. Schumacher J. Porquier A. Simon A. (2016). “Regulation of secondary metabolism in the gray mold fungus *Botrytis cinerea*,” in *Host-Pathogen Interaction: Microbial Metabolism, Pathogenicity and Antiinfectives*, eds Unden G. Thines E. Schüffler A. (Weinheim: Wiley-VCH Verlag GmbH), 201–216. 10.1002/9783527682386

[B63] von Bargen K. W. Niehaus E.-M. Krug I. Bergander K. Würthwein E. U. Tudzynski B. (2015). Isolation and structure elucidation of fujikurins A-D: products of the PKS19 gene cluster in *Fusarium fujikuroi*. *J. Nat. Prod.* 78 1809–1815. 10.1021/np5008137 26192387

[B64] Weber T. Kim H. U. (2016). The secondary metabolite bioinformatics portal: computational tools to facilitate synthetic biology of secondary metabolite production. *Synth. Syst. Biotechnol.* 1 69–79. 10.1016/j.synbio.2015.12.002 PMC5640684 29062930

[B65] Wiemann P. Albermann S. Niehaus E.-M. Studt L. von Bargen K. W. Brock N. L. (2012). The Sfp-type 4’-phosphopantetheinyl transferase Ppt1 of *Fusarium fujikuroi* controls development, secondary metabolism and pathogenicity. *PLoS ONE* 7:e37519 10.1371/journal.pone.0037519 PMC3360786 22662164

[B66] Wiemann P. Brown D. W. Kleigrewe K. Bok J. W. Keller N. P. Humpf H. U. (2010). FfVel1 and FfLae1, components of a velvet-like complex in *Fusarium fujikuroi*, affect differentiation, secondary metabolism and virulence. *Mol. Microbiol.* 77 972–994. 10.1111/j.1365-2958.2010.07263.x 20572938 PMC2989987

[B67] Wiemann P. Sieber C. M. K. von Bargen K. W. Studt L. Niehaus E.-M. Espino J. J. (2013). Deciphering the cryptic genome: genome-wide analyses of the rice pathogen *Fusarium fujikuroi* reveal complex regulation of secondary metabolism and novel metabolites. *PLoS Pathog.* 9:e1003475 10.1371/journal.ppat.1003475 PMC3694855 23825955

[B68] Wiemann P. Willmann A. Straeten M. Kleigrewe K. Beyer M. Humpf H.-U. (2009). Biosynthesis of the red pigment bikaverin in *Fusarium fujikuroi*: genes, their function and regulation. *Mol. Microbiol.* 72 931–946. 10.1111/j.1365-2958.2009.06695.x 19400779

[B69] Yin W. Keller N. P. (2011). Transcriptional regulatory elements in fungal secondary metabolism. *J. Microbiol.* 49 329–339. 10.1007/s12275-011-1009-1 21717315 PMC3714018

[B70] Zhuang Z. Lohmar J. M. Satterlee T. Cary J. W. Calvo A. M. (2016). The master transcription factor *mtfA* governs aflatoxin production, morphological development and pathogenicity in the fungus *Aspergillus flavus*. *Toxins* 8:29 10.3390/toxins8010029 PMC4728551 26805883

