# Erratum: The GATA-Type Transcription Factor Csm1 Regulates Conidiation and Secondary Metabolism in *Fusarium fujikuroi*

**DOI:** 10.3389/fmicb.2017.01846

**Published:** 2017-09-14

**Authors:** Frontiers Production Office

**Affiliations:** Frontiers Media SA Lausanne, Switzerland

**Keywords:** *Fusarium fujikuroi*, GATA transcription factor, conidiation, secondary metabolism, gene expression, NsdD, Csm1, STC

The publisher incorrectly published an article which included Eduard Spitzer as an author. He has now been removed and the publisher apologizes for this error.

The original article has been updated.

