# Supplementary material for: The GATA-Type Transcription Factor Csm1 Regulates Conidiation and Secondary Metabolism in Fusarium fujikuroi
Source: Front Microbiol. 2017 Jun 26;8:1175. doi: 10.3389/fmicb.2017.01175 (PMC5483468; doi:10.3389/fmicb.2017.01175)
Supplement: Supplementary file 1 [file Table_1.DOCX]

Table S1: Oligonucleotide primers used in this work

| **Primer name** | **Sequence 5' - 3'** |
| --- | --- |
| *CSM1*-sR1 | CCTCTGGGAATGATCGAGGAGC |
| Glu-term-F2 | GCGGCCGCTTAGCGTATGTAGATAAGATGTATG |
| hphF-trpC-P | GTCGGAGACAGAAGATGATATTGAAGGAGC |
| hphF-trpC-T | GTTGGAGATTTCAGTAACGTTAAGTGGAT |
| hphR-trC-T2 | GTTGGAGATTTCAGTAACGTTAAGTGGATCGTATCTTATCGAGATCCTGAACACC |
| nat1-seqF1 | CGGACGGCGAGCGGCAGGCGC |
| *CSM1*_comp-TerR | CCAATTCGGAACACCAGATACACC |
| *CSM1*_intron-diag-2 | ATGCCACCACATATCCCTGG |
| *CSM1*_seq-1 | AGTCTTGATAGTTCCAGC |
| *CSM1*_seq-2 | TGGGCGATTCTGCTGTAAGC |
| *CSM1*-3F-diag | CACTGATCCCTCCAAAGCGC |
| *CSM1*-3F | CTCCTTCAATATCATCTTCTGTCTCCGACACTCTTCCCGCCACGGTGGG |
| *CSM1*-3R | GCGGATAACAATTTCACACAGGAAACAGCTCATCAATGATGTCTCCGCC |
| *CSM1*-5F-diag | CCAGCCGTGGCTCAAGGCCG |
| *CSM1*-5F | GTAACGCCAGGGTTTTCCCAGTCACGACGTCGACATCGCTGTCAACGGG |
| *CSM1*-5R | ATCCACTTAACGTTACTGAAATCTCCAACGTTCTCGATACGACCTGTCC |
| *CSM1*-WT-F | CACAACCCAGCTCTGGTGC |
| *CSM1*-WT-R | GCGTAATGCAACCCGCAGG |
| pCSN44-hph-trpC-T | GGAATAGAGTAGATGCCGACCGG |
| pCSN44-trpC-P2 | GTGATCCGCCTGGACGACTAAACC |
| PoliC-seqF2 | GGGAGACGTATTTAGGTGCTAGGG |
| Tgluc-seq-R2 | CCGCCCTCTTTTGTCTTCCGC |
| BcGluc-T-seqR1 | GGGTCCATGCTAATACTTATGTAC |
| Tgluc-nat1-R | CCACTTAACGTTACTGAAATCTCCAACATCTTGTTGGGGGGAAGGGGT |
| *oGFP*-F | ATGGTTTCCAAGGGTGAGG |
| *oGFP*-NotI-R1 | CATACATCTTATCTACATACGCTAAGCGGCCGCTTTGTAAAGTTCATCCATTCCC |
| *CSM1*-mut-F | CAAATGGCCAGGCGCTGCCCGCAATCTCG |
| *CSM1*-mut-R | CGAGATTGCGGGCAGCGCCTGGCCATTTG |
| *CSM1*-comp-5'-*LTF1*-F | GGACAGGTCGTATCGAGAACCTCCCGCCATGGAGGGTGCGGAAAGTGG |
| *LTF1*-Tgluc-R | CATACATCTTATCTACATACGCTATGACCGTGGTGAATGATCC |
| *CSM1*-Tgluc-R | TAATCATACATCTTATCTACATACGTCATTGTCGATGAGCCTCATC |
| *CSM1*-com-GFP-R | TACTTACCTCACCCTTGGAAACCATTTGTCGATGAGCCTCATCTTTAGG |
| *CSM1*-com-F | TTGGACAGGTCGTATCGAGAACCTCCCGCCATGGCAACGGCAACCCTCATC |
| *CSM1-ltf1*-F | AGTAAAATATTCCCAGATCTTACAATGGCAACGGCAACCCTCATC |
| *CSM1-ltf1*-R | CATACATCTTATCTACATACGTCATTGTCGATGAGCCTCATC |
| *LTF1*-hi5F | CTCACGAAACGCACCCTACGATTC |
| *LTF1*-hi3R | CAGCGCACAGCGCATAGTGCATAC |
| *Nat1*-hiR | GGTAAGCCGTGTCGTCAAGAG |
| *Hph*-hiF | GTCTGGACCGATGGCTGTGTAGAAG |
| PR086f_WP030314776 | ATGCCTCAGCCCTTCGTTATGC |
| PR086r_WP030314776 | TCAGACCCTGACCGGCC |
| PR088f_WP030314776 | GGCAGCCATATGGCTAGCATGACTGGTGGAATGCCTCAGCCCTTCGTTATGC |
| PR088r_WP030314776 | TCTCAGTGGTGGTGGTGGTGGTGCTCGAGTTCAGACCCTGACCGGCC |
| Invitro_STC1_F | GGCAGCCATATGGCTAGCATGACTGGTGGAATGCAGGTGTTCACTACCAATATGG |
| Invitro_STC1_R | TCTCAGTGGTGGTGGTGGTGGTGCTCGAGTTCAACCTTTCAGAATGGTAAAATAAAAAGC |
| *BIK2*-F | CTTGAGTCTGATAGAGGCGC |
| *BIK2*-R | ACGGCGCAGCAGAAAGTGCC |
| *FSR2*-F | TGTCATTGAGCATGCTACGC |
| *FSR2*-R | ATCAGCCTTGGTAAGCAGGG |
| *CSM1*-F | CACAACCCAGCTCTGGTGC |
| *CSM1*-R | GCGTAATGCAACCCGCAGG |
| *LTF1*-F | ATGGAGGGTGCGGAAAGTGGC |
| *LTF1*-R | CTATGACCGTGGTGAATGATCC |
| *LTF2*-F | ATGTCACACACATCAGGCAA |
| *LTF2*-R | TTAGTACAGCCTCTCTCCAAAAGC |
| *PKS13*-F | GAGAACCCACTTGCATACTTAG |
| *PKS13*-R | CCGTGGTGACATGTTGAAGAATC |
| *CCG1*-F | GGAGACTGTCAAGGTAAGCC |
| *CCG1*-R | CTAGTGCTTGGCAGCCTCTG |
