# Supplementary material for: The GATA-Type Transcription Factor Csm1 Regulates Conidiation and Secondary Metabolism in Fusarium fujikuroi
Source: Front Microbiol. 2017 Jun 26;8:1175. doi: 10.3389/fmicb.2017.01175 (PMC5483468; doi:10.3389/fmicb.2017.01175)
Supplement: Supplementary file 4 [file Image_1.PDF]

Supplementary

The GATA-type transcription factor Csm1 regulates conidiation and secondary metabolism in *Fusarium fujikuroi*

Eva-Maria Niehaus[1Ψ], Julia Schumacher[1], Immo Burkhardt[2], Patrick Rabe[2], Eduard Spitzer[1], Martin Münsterkötter[3], Ulrich Güldener[4], Christian M. K. Sieber[5], Jeroen S. Dickschat[2], Bettina Tudzynski[1]*

[1]Institut für Biologie und Biotechnologie der Pflanzen, Westfälische Wilhelms-Universität Münster, Münster, Germany
[2]Kekulé-Institut für Organische Chemie und Biochemie, Rheinische Friedrich-Wilhelms-Universität Bonn, Bonn, Germany
[3]Institute of Bioinformatics and Systems Biology, Helmholtz Zentrum München, German Research Center for Environmental Health (GmbH), Neuherberg, Germany
[4]Department of Genome-oriented Bioinformatics, Wissenschaftszentrum Weihenstephan, Technische Universität München, Freising, Germany
[5]Department of Energy Joint Genome Institute, Walnut Creek, California, United States of America
[Ψ]Present address: Institute of Food Chemistry, Westfälische Wilhelms-Universität Münster, Münster, Germany

CORRESPONDENCE:
Prof. Dr. B. Tudzynski
tudzynsb@uni-muenster.de

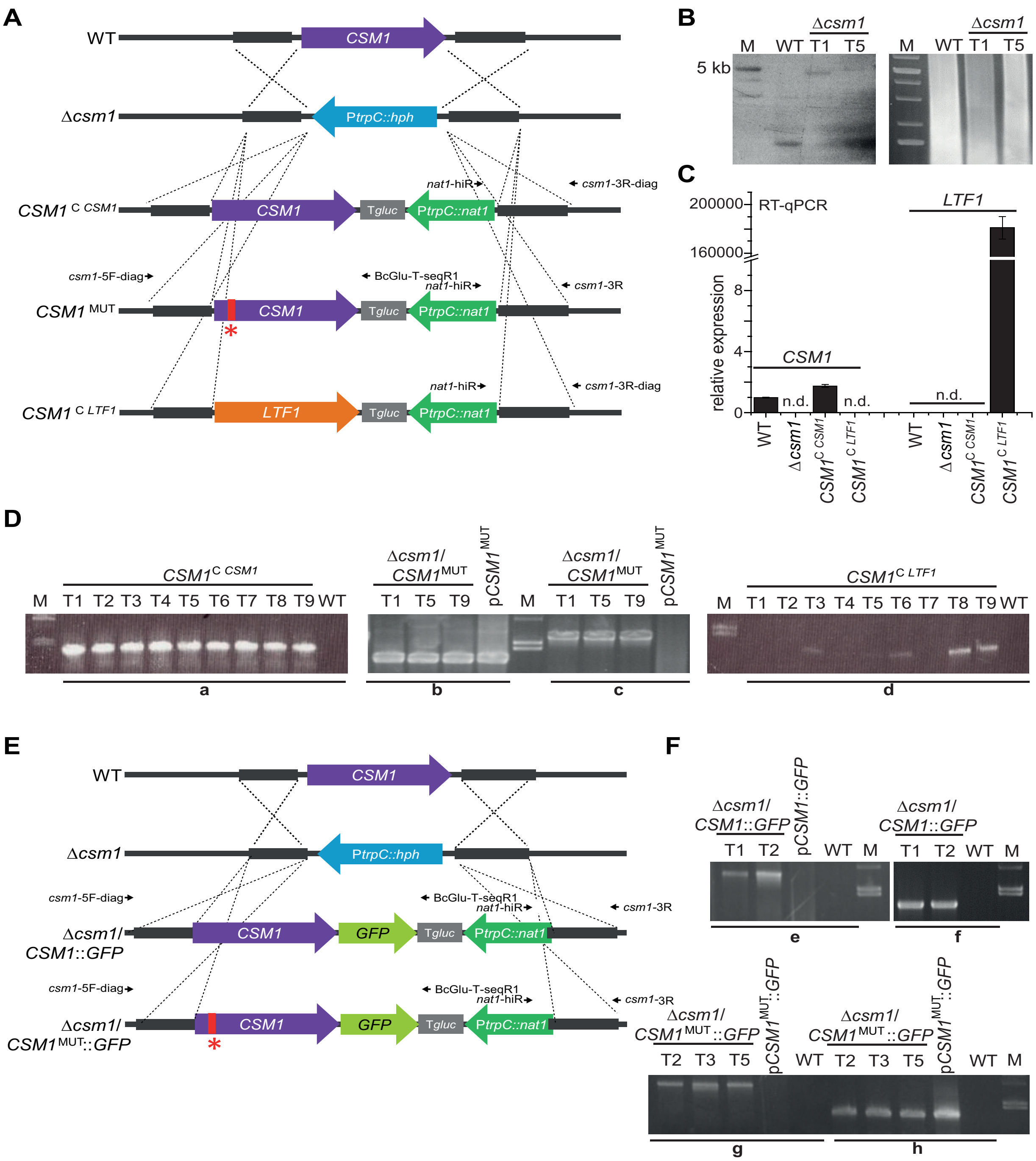

Figure S1: Strategies for functional analysis of *CSM1* and the conserved RQSLPSI motif. (A) All fragments were introduced into the *CSM1* locus of the Δ*csm1* mutant by homologous recombination. The red asterisk shows the change of three nucleotides of the putative motif 5'-CGCCAGTCGCTGCCCTCAATC-3' (RQSLPSI) into the following sequence: 5'-GGCCAGGCGCTGCCCGCAATC-3' (GQALPAI). **(B)** Southern blot of the WT and Δ*csm1* strains T1 und T5. Genomic DNA was digested with *Hin*dIII resulting in hybridizing fragments of 1.4 kb (WT) and 4.6 kb (Δ*csm1*), M = Gene Ruler 1 kb Plus Ladder. **(C)** Gene expression of *CSM1* and *LTF1* in WT, Δ*csm1*, and complemented strains. RT-qPCR was performed using cDNA derived from cultures grown for three days in ICI medium supplemented with 6 mM glutamine. The level in the WT was defined as 1. Mean value and standard deviation are derived from two replicates. **(D)** Diagnostic PCR of $CSM1^{C\ CSM1}$ mutants, a: nat1-hiR + csm1-3R-diag (1.6 kb), T1 and T2 were chosen for further experiments; diagnostic PCR of Δ*csm1*/$CSM1^{MUT}$, b: nat1-hiR + csm1-3R (1.5 kb) c: csm1-5F-diag + BcGlu-T-seqR1 (2.6 kb); diagnostic PCR of $CSM1^{C\ LTF1}$, d: nat1-hiR + csm1-3R-diag (1.6 kb), T6 and T8 were chosen

for further experiments; M = λ/*Hin*dIII (4.4, 2.3 and 2.0 kb). **(E)** Strategy for insertion of *GFP* constructs. **(F)** Diagnostic PCR of Δ*csm1*/*CSM1*::*GFP*, e: csm1-5F-diag + BcGlu-T-seqR1 (3.3 kb), f: nat1-hiR + csm1-3R (1.5 kb); diagnostic PCR of Δ*csm1*/*CSM1*^{MUT}::*GFP*, g: csm1-5F-diag + BcGlu-T-seqR1 (3.3 kb) h: nat1-hiR + csm1-3R (1.5 kb); M = λ/*Hin*dIII (4.4, 2.3 and 2.0 kb).

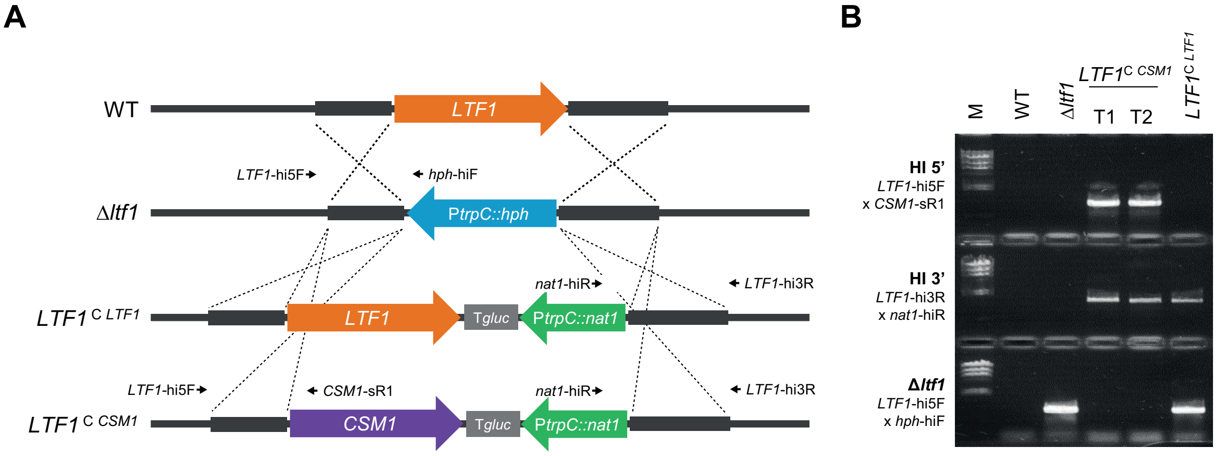

Figure S2: Integration of *CSM1* at the *B. cinerea LTF1* locus. (A) Strategy of mutant construction. *CSM1* was introduced at *LTF1* by replacement of the hygromycin resistance cassette according to the strategy used for the complementation of the Δ*ltf1* mutant (*LTF1*$^{C\ LTF1}$) in a previous study. **(B)** Diagnostic PCR confirmed the targeted integration of *CSM1*. Primer combinations as indicated were used to detect the homologous integration at 5' and 3' of *LTF1*. Furthermore, the homokaryotic status of the *LTF1*$^{C\ CSM1}$ mutants (T1, T2) was demonstrated (absence of Δ*ltf1* allele).

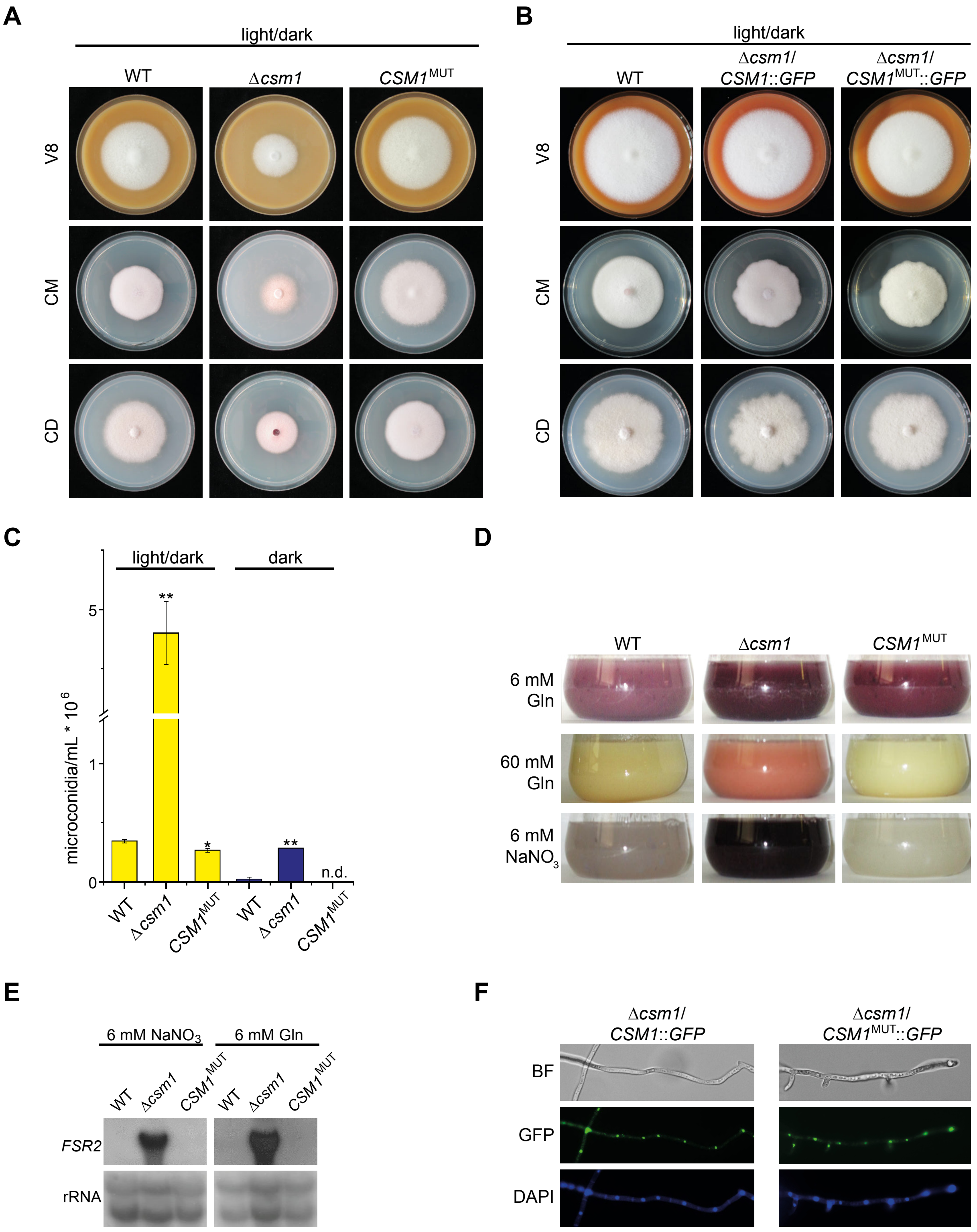

Figure S3: Complementation of the Δ*csm1* mutant with the mutated *CSM1* copy. (A) Colony morphology after 5 d. **(B)** Colony morphology after 6 d. **(C)** Number of microconidia produced in light/dark (12h/12h) and in the dark. Mean values and standard deviations shown derived from three biological replicates. Statistical tests (t-test) revealed significant differences (*p < 0.05, **p < 0.001). **(D)** Pigmentation of the cultures. **(E)** Northern blot analysis of WT, Δ*csm1* and *CSM1*^{MUT} grown in 6 mM $NaNO_3$ or 6 mM glutamine (Gln) for 2 d. The *O*-methyltransferase *FSR2* was used as probe. **(F)** The subcellular localization of the Csm1 and Csm1^{MUT}-GFP fusion constructs by fluorescent microscopy (GFP). Nuclei were visualized by using Hoechst solution for staining (DAPI); BF = bright field.

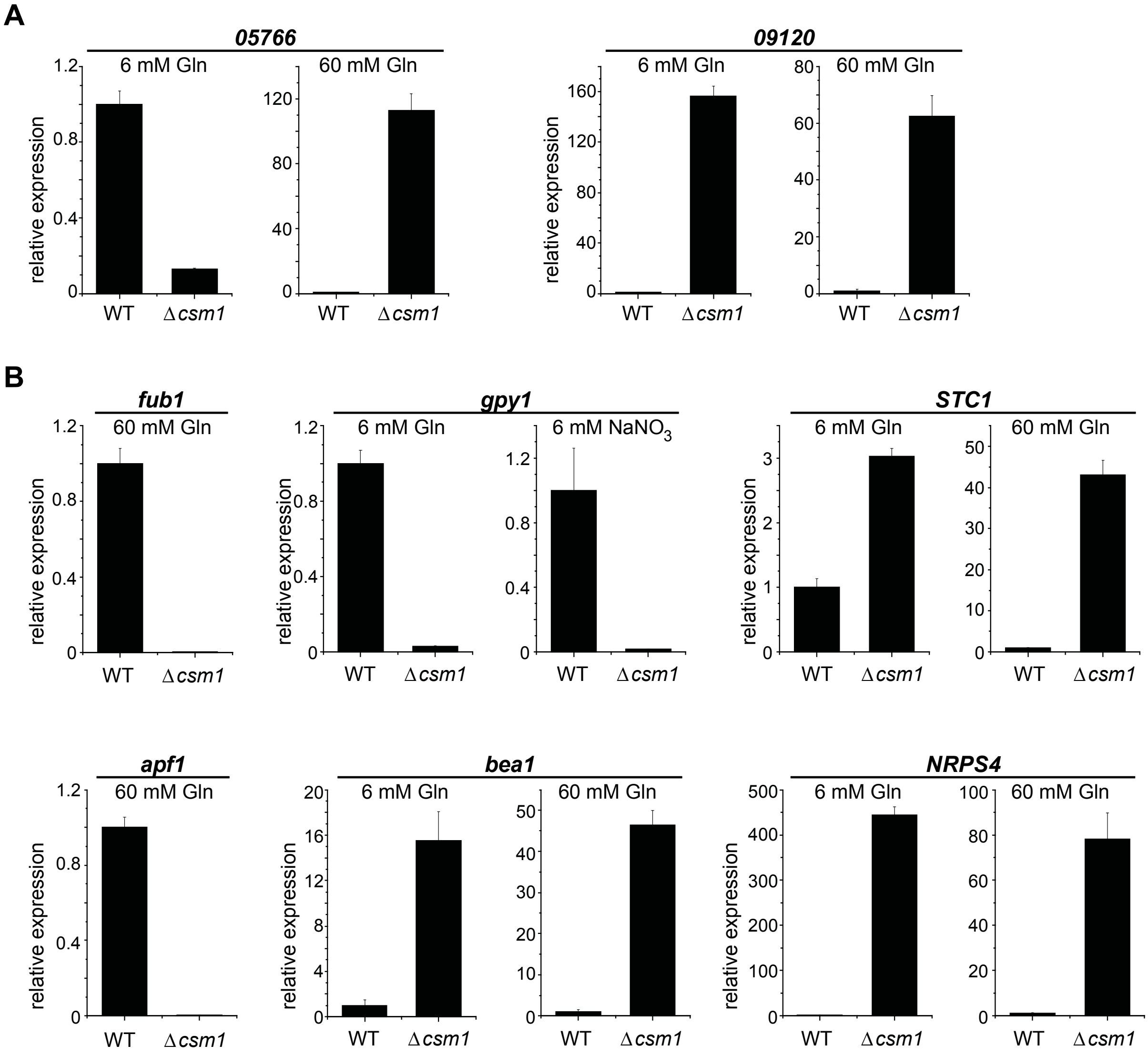

Figure S4: Quantitative real-time PCR (qRT-PCR) analysis of the relative expression of some potential Csm1 target genes using the ΔΔCt method. The WT and the Δ*csm1* mutant were cultivated for 2 d in ICI medium supplemented with either 6 mM or 60 mM glutamine (Gln) or 6 mM $NaNO_3$. Total RNA from the harvested mycelium was used for cDNA synthesis. Error bars (±SD) originate from a technical replicate and expression of the WT was arbitrarily set to 1. **(A)** Expression analysis of the randomly chosen Csm1-dependent transporter genes FFUJ_05766 and FFUJ_09120 encoding an ABC transporter and a putative 4-hydroxybenzoate transporter, respectively (see Table S2). **(B)** Expression analysis of some secondary metabolite key enzyme-encoding genes: *fub1* – encoding the polyketide synthase (PKS) of fusaric acid, *gpy1* – encoding the PKS of gibepyrone, *STC1* – encoding the sesquiterpene cyclase of the putative STC1 cluster, *apf1* – encoding the non-ribosomal peptide synthetase (NRPS) of apicidin F, *bea1* – encoding the NRPS of beauvericin, *NRPS4* – encoding the NRPS of the putative NRPS4 cluster.

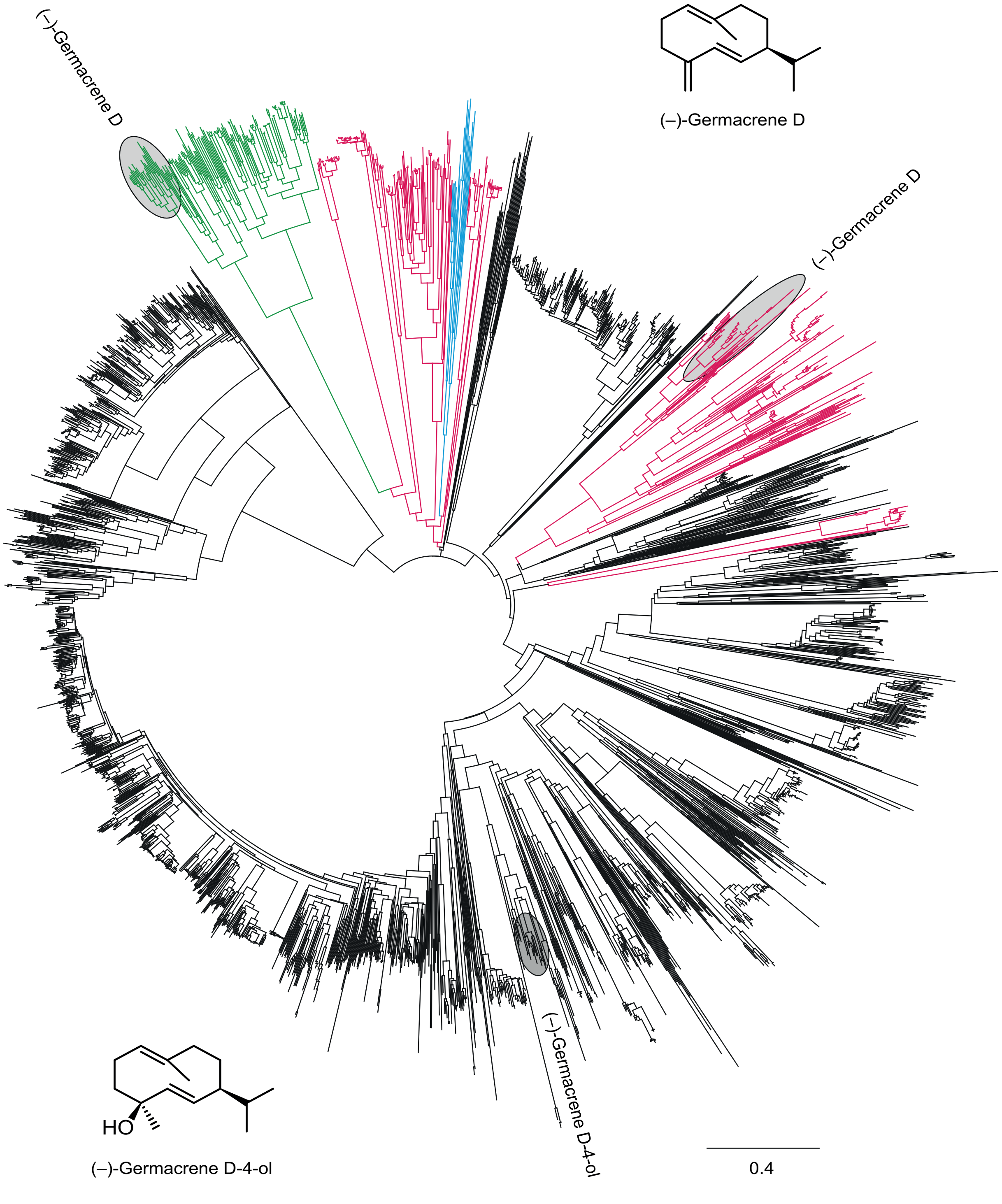

Figure S5: Phylogenetic tree of 2500 terpene cyclase orthologs. The green branches represent plant enzymes, the red branches are fungal enzymes, bacterial enzymes are in black, and the blue branches represent the recently discovered terpene cyclases from social amoebae. The known (–)-germacrene D synthases from plants and fungi and the (–)-germacrene D-4-ol synthases from bacteria are highlighted.
